# The Devastating Rice Blast Airborne Pathogen *Magnaporthe oryzae*—A Review on Genes Studied with Mutant Analysis

**DOI:** 10.3390/pathogens12030379

**Published:** 2023-02-26

**Authors:** Jinyi Tan, Haikun Zhao, Josh Li, Yihan Gong, Xin Li

**Affiliations:** 1 Michael Smith Laboratories, University of British Columbia, Vancouver, BC V6T 1Z4, Canada; 2 Department of Botany, University of British Columbia, Vancouver, BC V6T 1Z4, Canada

**Keywords:** plant fungal pathogen, rice blast, *Magnaporthe oryzae*, mutant analysis, knockout

## Abstract

*Magnaporthe oryzae* is one of the most devastating pathogenic fungi that affects a wide range of cereal plants, especially rice. Rice blast disease causes substantial economic losses around the globe. The *M. oryzae* genome was first sequenced at the beginning of this century and was recently updated with improved annotation and completeness. In this review, key molecular findings on the fungal development and pathogenicity mechanisms of *M. oryzae* are summarized, focusing on fully characterized genes based on mutant analysis. These include genes involved in the various biological processes of this pathogen, such as vegetative growth, conidia development, appressoria formation and penetration, and pathogenicity. In addition, our syntheses also highlight gaps in our current understanding of *M. oryzae* development and virulence. We hope this review will serve to improve a comprehensive understanding of *M. oryzae* and assist disease control strategy designs in the future.

## 1. Introduction

*Magnaporthe oryzae* (synonym of *Pyricularia oryzae*), which causes rice blast disease, is a filamentous fungus belonging to the Ascomycota phylum. In 1879, it was initially named *Trichothecium griseum* by Cooke without a detailed description [1]. In the following century, several names were given to the fungus based on the teleomorph and anamorph stages, such as *Ceratosphaeria grisea* by Herber (1971) and *Dactylaria oryzae* by Sawada (1917). In 1990, based on host specificity, physiological differences, and genetic evidence, Rossman corrected its name to *Pyricularia oryzae* [2]. *P. oryzae* was used to refer to the asexual stage, and the sexual stage was named *Magnaporthe grisea* by Couch and Kohn (2002). Additional phylogenetic analysis and interstrain fertility tests showed that *M. grisea* should be assigned to *Digitaria* (crabgrass)-infecting isolates, whereas *M. oryzae* causes damage to rice and other important crops in the *Poaceae* family, such as millet and wheat [3].

*Magnaporthe oryzae* is one of the most devastating agricultural pathogens since it primarily attacks cultivated rice (*Oryza sativa*), an important staple food feeding over 50% of the world’s population [4,5]. Its infection can be destructive under favorable conditions, contributing to 10–30% of the annual rice yield loss [6,7]. Given that it can affect both temperate and tropical rice growth, the disease has been widely distributed in 85 countries with various environmental conditions [7,8,9]. As a consequence, it is ranked the most damaging fungal pathogen in the world [9].

*Magnaporthe oryzae* can cause disease on rice plants at all developmental stages and can infect different tissues, including leaves, stems, nodes, and panicles [10]. Its aerial conidiophores produce three-celled and teardrop-like conidia that are arranged in a sympodial manner. The attachment of these conidia to the host surface initiates the infection cycle [11]. Conidia adhere to the host with the help of mucilage, a thick and gluey substance stored at the spore tip. They then germinate and generate germ tubes before appressoria differentiation [10,11,12]. The death of three-celled conidia triggers the formation of single-celled appressoria [13]. Then, appressoria become melanized and accumulate glycerol, generating turgor to create pressure for penetration into the host epidermal cells [13]. Upon penetration, it colonizes plants using invasive hyphae that grow intracellularly [14]. Notably, membrane caps, formed on the invading hyphal tips, are used for cell-to-cell movement via manipulating the plasmodesmata [14]. Thus, the membrane interactions between *M. oryzae* and plant hosts are important for the invasion of hyphae into plant cells [15].

Upon penetration, fungal pathogens can secret effectors to promote virulence and suppress host immunity [16]. In turn, plants have evolved resistance genes (R) that encode R proteins to recognize the presence of effectors and enable effector-triggered immunity (ETI) [16,17]. In *M. oryzae*, when the first hyphal cell invades, effectors accumulate in the biotrophic interfacial complex (BIC), the site for effector delivery [15,18]. Two types of effectors were defined in *M. oryzae* based on their localization in plant cells: cytoplasmic effectors entering the host cell cytoplasm via the BIC, and BIC-independent apoplastic effectors being delivered into the apoplast [19,20].

Due to its notable appressoria formation, the secretion of effectors during the invasion, and the effector delivery to the apoplast and cytoplasm of plant cells, *M. oryzae* has been used as a model plant fungal pathogen to understand pathogen genomics and pathogen–host interactions [11]. Focusing on the biological development and pathogenesis of *M. oryzae*, the current key molecular findings are summarized in this review, as well as its genomic features. Overall, this review primarily aims to build a clear and comprehensive understanding of the genetic and molecular mechanisms underlying its biology. Such an understanding will provide insight into potential strategies to reduce the economic losses caused by it. We also hope that this review can aid in the study of other fungal pathogens.

## 2. The Features of *M. oryzae* Genome

### 2.1. Genome Sequencing

The first analysis of the *M. oryzae* genome was published (as *M. grisea*) in 2005 using the whole-genome shotgun (WGS) approach, with 7× coverage. The genome of *M. oryzae* 70-15, originally derived from a cross between isolates of rice and weeping lovegrass, consisted of seven chromosomes with an estimated size of 38.8 Mb (GenBank accession number: AACU00000000.3) [21,22]. However, this draft genome sequence was compromised because of significant retrotransposon content. After improvements in verifying unique sequence anchors, extending contigs and scaffolds, and filling the remaining gaps, the genome sequence of the *M. oryzae* strain 70-15 was refined at the Broad Institute in 2015 with an estimated size of 41 Mb. The high-confidence annotation and gene models were also generated using multiple methods including RNA-seq data and expressed sequence tags (ESTs) alignments, and homologous gene and Basic Local Alignment Search Tool (BLAST) searches [23]. Therefore, this 41 Mb genome sequence of strain 70-15 became the standard reference for *M. oryzae*, consisting of seven chromosomes with about 51.6% GC content (BioProject Accession number: PRJNA13840).

The initial analysis of the *M. oryzae* strain 70-15 sequenced in 2005 revealed a family of G-protein-coupled receptors that are specific to *M. oryzae* and have an expression in infection-related development [22]. Furthermore, they also unveiled three mitogen-activated protein kinase (MAPK) cascades including appressoria development and penetration peg formation, which are crucial in the plant infection process, as well as the relevant genes involved in these signaling cascades. In addition, a significant number of predicted secretion proteins and putative effector proteins, as well as synthases/synthetases involved in secondary metabolic pathways, were identified [22]. Many of these became targets for reverse genetic analysis.

Subsequently, the genome sequences of many other *M. oryzae* strains became available. For example, Xue et al. (2012) [24] sequenced two *M. oryzae* field isolates, Y34 (isolates from China) and P131 (from Japan), using Sanger and 454 sequencing. Genome comparisons revealed that the genome size and overall structure were similar among different strains, but more genes that might be involved in host–pathogen interactions were found in the field isolates [24]. They also found different distributions of transposon-like elements among these three strains, indicating genetic variations [24]. More recently, Dr. T. R. Sharma’s group sequenced the whole genome of two isolates from India, an avirulent isolate RML-29 and a highly virulent isolate RP-2421, and identified several isolate-specific genes and potential effectors through pan-genome analysis, which might be involved in fungal pathogenicity and fungal–plant interactions [25,26].

Repetitive transposable elements (TEs) may play important roles in both genome and pathogenic variations in *M. oryzae* [22,24,27]. They may mediate fungal virulence via the inactivation or deletion of pathogen-associated molecular patterns (PAMPs)-encoding genes and can trigger plant defense responses [28,29]. The *M. oryzae* genome contains approximately 10% of the repetitive sequences in various isolates [22,23,24,27]. Xue et al. (2012) [24] also uncovered that 23.8% of the TE-disrupted genes were predicted to encode signal peptide sequences, highlighting that this percentage was higher than the average percentage of the whole genome. The single-molecule real-time sequencing also revealed that TEs are involved in chromosomal translocation and secreted proteins (SPs) polymorphisms [27]. In addition, transposon and effector-rich mini-chromosomes were observed in the *M. oryzae* MoT isolate, which contribute to the field adaptation [30]. These findings suggested a significant role of TEs in defining host specificity and fungal virulence.

### 2.2. Transcriptomic and Secretome Analysis

The availability of whole-genome sequencing data has facilitated the transcriptomic and secretome analysis of *M. oryzae*. The first comprehensive genome-wide transcriptional profiling study of *M. oryzae* was carried out using microarray analyses. Changes in genome-wide gene expression during the early stages of spore germination and appressoria formation were identified [31]. A microarray analysis of the fungal-invading hyphae at an early stage (36 h post inoculation) uncovered several putative effectors, including fungal biotrophy-associated secreted (BAS) proteins [20]. Moreover, microarray data of invasive growth on rice and barley at a later stage (72 hpi) also revealed multiple genes associated with stress responses and invasive growth [32].

In recent years, high-throughput RNA sequencing has become an efficient approach to provide high-quality transcriptome data. Many transcriptomic analyses were carried out to study host–pathogen interactions. For example, Kawahara et al. (2012) [33] investigated the mixed transcriptome of the pathogen *M. oryzae* and host *O. sativa*, revealing 240 upregulated genes encoding potential secreted proteins and many known infection-related genes in *M. oryzae* [33]. Another study on plant–fungal interactions between the host rice plants and *M. oryzae* discovered several novel effectors and virulence-related genes, including 98-06 isolate-unique genes IUG6 and IUG9, which were involved in the fungal pathogenicity and located in the BIC [34].

Secretome analysis is another useful approach to identifying crucial genes through studying secreted proteins and their secretion pathways. One *M. oryzae* secretome study during the early stages of infection identified 53 secreted proteins, including proteins that functioned by modifying fungal lipid and cell walls, detoxifying reactive oxygen species, as well as encouraging fungal metabolism [35]. Liu et al. (2021) [36] also identified several secreted proteins requiring N-glycosylation, which play essential roles in fungal pathogenicity and cell wall integrity [36]. Combining transcriptomic and secretome analyses, these datasets provide an abundant reservoir of candidates for reverse genetic analysis to help understand the molecular mechanisms in *M. oryzae*–rice interactions.

**Figure 1:**
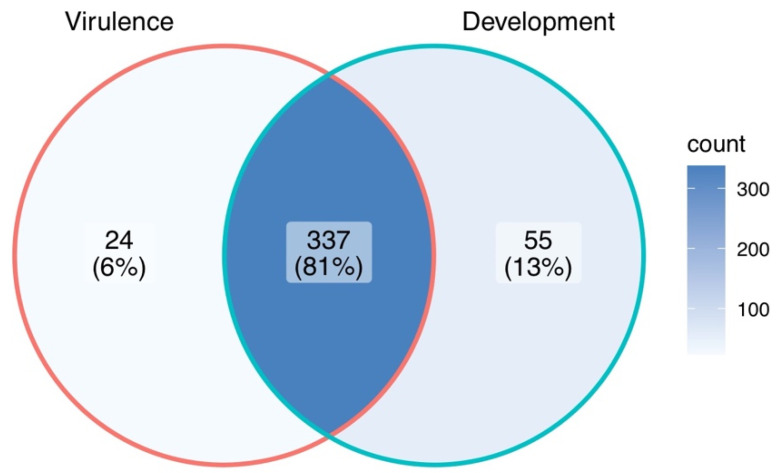
A Venn diagram summary of *M. oryzae* genes studied with mutant analysis (detailed in Supplementary Table S1).

**Figure 2:**
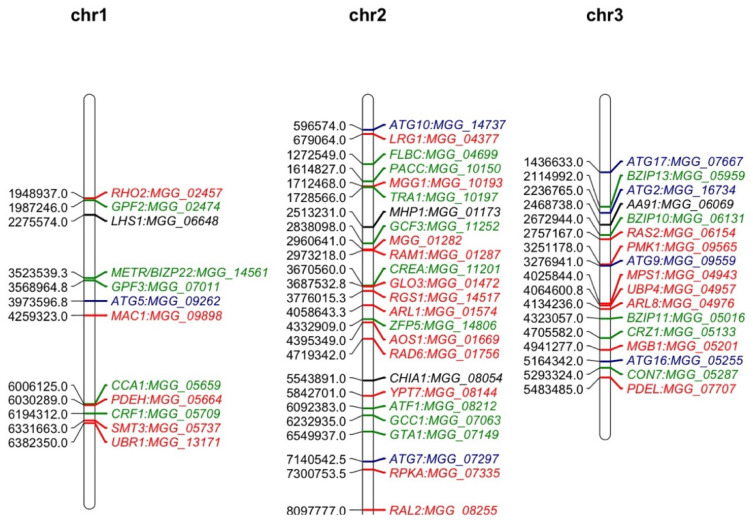

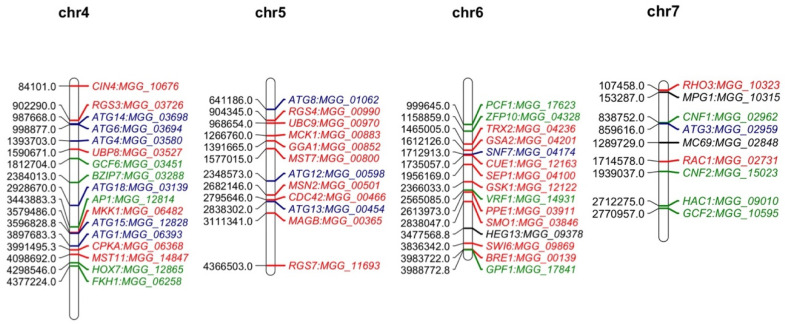
Map positions of important genes studied in the *M. oryzae* genome through mutant analysis. The numbers on the left of each chromosome represent the locations of these genes. Genes in this map contribute to both development and virulence of *M. oryzae*. Genes labeled in red are components in different important signaling pathways. Genes labeled in green are transcription factor-encoding genes. Genes labeled in blue are autophagy related while the ones in black are genes encoding effectors. The chromosomal map was drawn using ‘MapChart’ software using information from Supplementary Table S1.

Mutant analysis is a definitive process used to establish a causal relationship between a gene and a specific biological process. Many methods were applied to generate mutants in *M. oryzae*, such as T-DNA insertion, RNA interference (RNAi), and homologous recombination (deletion). Among them, gene deletion via homologous recombination has been the most commonly used method in the past few decades. The latest CRISPR technology has also been developed to improve gene-editing efficiency [37,38]. The rest of this review will give an overview of all *M. oryzae* genes studied to date using such approaches (Supplementary Table S1). A Venn diagram of the listed genes is provided for readers’ overview (Figure 1). Over 400 genes have been summarized in this review, and it is clear to see that most of them contribute to both the development and virulence of *M. oryzae*. A chromosomal map including the significant genes involved in both processes was generated to detect possible cluster patterns and genomic features (Figure 2).

## 3. Molecular Dissection of *M. oryzae* Biology

In the past, various gene/mutant/protein nomenclatures were used by *Magnaporthe* researchers. Here we adopt the most commonly used one for this review. For example, the wild-type gene is abbreviated as italicized capital letters (*ABC1*), while the mutant is indicated with lower case letters (*abc1*). The protein it encodes is presented in capital letters (ABC1). Exceptions will be explained. In addition, it is important to bear in mind that huge variations exist in different field isolates/strains and plant hosts among these studies, contributing to the discrepancies that are sometimes observed in mutant phenotypes of the same genes.

### 3.1. Genes Mainly Related to Fungal Development

In the earlier years of molecular studies, scientists focused on the more obvious morphological and physiological traits of *M. oryzae* (Supplementary Table S1a). Similar to most fungi, asexual spores of *M. oryzae*, also known as conidia, play an important role in its life cycle. Conidia are produced from aerial conidiophores in a sympodial arrangement, and their production and dissemination serve as the major source of inoculum for *M. oryzae* [11]. Lee et al. (2006) [39] revealed that conidia formation was light-dependent and blue light inhibited the asexual development of *M. oryzae*. A mutant *mgwc-1* was also identified, which is the homolog of the blue light receptor White Collar-1 of *Neurospora crassa*, showing a delayed conidia formation [39]. Thus, the light condition is a major cue for conidiation induction.

A T-DNA insertion mutant showing conidiation deficiency revealed that conidiophore stalk-less 1 (COS1), a Cys2-His2 (C2H2) zinc-finger protein, plays a determining role in *M. oryzae* conidiation [40]. The *cos1* mutant affects two conidiophore-related genes while causing similar symptoms compared to WT in root and foliar infection assays [40]. Such results indicate that COS1 is a transcriptional regulator involved in conidiation but is not required for fungal virulence [40]. A C3HC-type zinc-finger domain protein interacting with the mitochondrial ATP-dependent Lon protease, ZFC3, was also shown to regulate mitochondrial genes and contribute to conidia production [41]. Through a gene disruption screen, Saitoh et al. (2014) [42] identified *ST1* encoding a sugar transporter in the hexose transporter family. Mutants with *ST1* deletion exhibited compromised conidiation and mycelial myelinization. However, its ability to cause blast disease on rice was unaltered, indicating a unique role of *ST1* on *M. oryzae* development [42]. Many other transcription factor (TF)-encoding genes, such as forkhead-box TF (HCM1) and homeobox TFs (HOX2 and HOX6), also only contribute to fungal development, which will be discussed later in the transcription factor section.

Chitin is an essential cell wall component for fungi that can be modified via deacetylation. Two chitin deacetylases (CDA1 and CDA4) can catalyze chitin deacetylation and influence *M. oryzae*’s vegetative growth but showed no effect on the fungal pathogenicity [43]. Mutants with *CDA1* deletion showed chitin deacetylation in mature hyphae, while the *cda4* mutants deacetylated chitin in young hyphae [43]. Lipid biosynthesis is also involved in *M. oryzae* development. The fatty acid synthase beta subunit dehydratase (FAS1) contributes to the conidiogenesis, pigmentation, and appressoria formation of *M. oryzae* [44]. A secondary metabolism regulator, LAEA (loss of AflR expression), which is involved in penicillin G biosynthesis, also functions in the fungal conidia production and sporulation [45].

Some other genes that only contribute to asexual development in *M. oryzae* have also been identified. For example, the calpains-related gene, *CAPN1*, is engaged in *M. oryzae* conidiation but not in fungal pathogenicity [46]. Additionally, the deletion of the spindle pole antigen gene *SPA2*, discovered from a mutant with defective colony and conidia formation, led to defects in hyphal growth and conidia production with normal pathogenicity [47]. Additionally, Lu et al. (2008) [48] discovered that the *MTP1* gene, encoding a type III integral transmembrane protein, was also required for the conidiation and conidial germination of *M. oryzae*. *mtp1* mutants exhibited delayed appressoria formation and similar virulence to WT, indicating that this gene is not necessary for fungal virulence [48]. Moreover, a class II histone deacetylase, HDA1, is also involved in fungal vegetative growth and conidiation [49].

In addition to the genes that regulate asexual development, several genes were found to affect sexual reproduction in *M. oryzae*. This fungus is heterothallic, where the formation of sexual structures (i.e., perithecia, asci, and ascospores) and the completion of its mating requires compatible partners with opposite mating types [50]. The *M. oryzae* loci that determine mating types were described as *MAT1-1* and *MAT1-2* to represent the opposite mating types. Each *MAT* gene has different functions. For example, *MAT1-1-1*, *MAT1-1-3*, and *MAT1-2-1* played important roles in perithecia development, and *MAT1-1-2* affected the formation of asci and ascospores [50]. However, *MAT1-2-2*, likely redundant with *MAT1-1-3*, was dispensable for sexual development [50]. Wang et al. (2021) [50] also found that mutants with *MAT* loci deletion showed no differences in vegetative growth, asexual development, and fungal virulence compared to WT. This suggests the independence of *M. oryzae* sexual reproduction from other biological processes.

### 3.2. Autophagy in Different Biological Processes of M. oryzae

During autophagy, intracellular molecules and organelles are degraded through engulfment and lysosome fusion. Recent molecular analysis in *M. oryzae* revealed that it plays critical roles in many facets of rice blast biology, including development of appressoria and pathogenicity (Supplementary Table S1b).

Autophagy-related (ATG) proteins participate in different stages of the autophagic process. A comparative genomic analysis between *M. oryzae* and *Saccharomyces cerevisiae* classified 23 *M. oryzae* ATG proteins into different groups based on their functions and interactions [51]. The first group was the ATG1 kinase complex, including ATG1, ATG13, ATG17, and ATG29, orchestrating autophagosome formation [51,52]. The second group formed the autophagosome, including ATG3, ATG4, ATG5, ATG7, ATG8, ATG10, ATG12, and ATG16. The autophagosome is a double-membrane vesicle, which is an indicator of autophagy [53]. The assembly of the autophagosome is initiated by the serine/threonine protein kinase ATG1, and the substrate targeting is mediated by a ubiquitin-like modifier ATG8 [13,52,53,54]. ATG8 is first modified by the ATG4 family, which contains an active cysteine residue (Cys206) to cleave the carboxyl terminus of ATG8 for autophagosome generation [55]. ATG6 and ATG14, classified in the class III phosphatidylinositol 3-kinase (PI3K) complex group, interact with ATG8 and function at the early stage of autophagosome formation [56]. The ATG9-associated vacuolar/lysosomal cytoplasmic recycling system functions in relocating the autophagy machinery [57]. It has been reported that ATG8 and ATG9 play partially overlapping roles, and the ATG9 cycling through multiple colonization sites requires the involvement of ATG1, ATG2, and ATG18 [57].

Many *M. oryzae* ATGs’ functions have been revealed by studying autophagic-deficient mutants. Single-gene deletions of *ATG1* to *ATG10*, as well as *ATG12*, *ATG13*, and *ATG15* to *ATG18* caused compromised fungal virulence or a nonpathogenic phenotype on rice seedlings, suggesting their key roles in fungal pathogenicity [13,51,52,53,54,55]. Meanwhile, the involvement of ATGs in hyphal growth, conidia, and appressoria development was also studied. For example, the *atg1* mutants showed normal appressoria formation but reduced appressorium turgor pressure, defective conidiation, and conidial germination [52]. The deletion of *ATG8* in *M. oryzae* inhibited its starvation-induced autophagy as well as conidia cell death during appressoria development [13]. Abnormal conidia production and dissemination from ATG-deficient strains, including *atg1*, *atg4*, *atg5*, *atg8*, and *atg9*, also indicated the importance of autophagy in cellular remodeling during sporulation [13,51,52,53,54,55,57,58]. In addition to the asexual effect, the autophagic process also contributes to *M. oryzae* sexual reproduction by meditating protoperithecia and perithecia production [52,55]. These results indicate the diverse roles of ATGs in the fungal development and pathogenicity of *M. oryzae*.

The genome-wide analysis also revealed six selective ATG genes (*ATG11*, *ATG24*, *ATG26*, *ATG27*, *ATG28*, and *ATG29*) that are related to pexophagy, mitophagy, or reticulophagy [51]. However, the single-gene deletion mutants of these selective ATG genes all showed normal conidia or appressoria autophagy and abilities to cause rice blast disease on rice seedlings, indicating that selective autophagy is not required for fungal pathogenicity meditated by appressoria [51].

Some proteins interacting with ATGs and/or participating in autophagic processes have also been studied. SGA1, a predicted vacuolar glucoamylase, acts synergistically with ATG8 to breakdown glycogen for energy supply, which is required for the onset of sporulation [54]. Veneault-Fourrey et al. (2006) [13] generated a mutant with *NIMA* deletion, which affected mitosis and prevented autophagic conidial cell death, resulting in defects in appressoria differentiation and infection-structure formation [13]. Furthermore, overproduction-induced pheromone-resistant protein 2 (OPY2), casein kinase (YCK1), and VPS9-containing protein (VPS9) were reported to be involved in autophagy by regulating ATG8 degradation, which contributes to fungal development and pathogenicity [59,60,61]. Notably, VPS9, as a guanine nucleotide exchange factor (GEF) activating the endosome marker VPS21, recruits VPS34 and the PI3-K complex (ATG6 and ATG14) to function in autophagosome formation [61]. In addition, a histone acetyltransferase HAT1 was found to regulate autophagy via the acetylation of ATG3 and ATG9, and the *hat1* mutants exhibited degraded appressorium turgor pressure and compromised pathogenicity [62]. Another histone acetyltransferase, GCN5, can acetylate ATG7 to repress autophagy, which plays an essential role in fungal development and pathogenicity [63].

Endosomes and autophagy are closely connected. VPS35, a component of the cargo-recognition complex, regulates the conidial autophagic cell death response and autophagosome biosynthesis [64]. The deletion mutants of the cargo-recognition subcomplex (VPS35, VPS26, and VPS29) showed defects in asexual development and pathogenicity [64]. The endosomal sorting complex required for transport (ESCRT) complex also plays an essential role in fungal endocytosis and autophagy. In *M. oryzae*, the genes encoding the subunits of the ESCRT-0 and -III subcomplexes were identified. The ESCRT-0 subcomplex deletion mutants, *hse1* and *vps27*, formed abnormal vacuoles and showed severe ATG8 lipidation, indicating their contributions to the fungal autophagic process [65]. In addition, the ESCRT-0 subcomplex was revealed to regulate both sexual and asexual development as well as the fungal ability to penetrate host plants [65]. SNF7 and IST1 are subunits of the ESCRT-III subcomplex that play important roles in fungal autophagy, cell wall integrity, and fungal development and pathogenicity [65,66]. Two endoplasmic-reticulum-associated degradation (ERAD) ubiquitin ligases, HRD1 and DER1, are involved in lipid metabolism and autophagy [67]. Deletion mutants of these two genes showed defects in conidial autophagic cell death, impaired appressoria development, and attenuated pathogenicity [67]. Many other genes, such as SNT2 encoding the SaNT domain-containing protein, HMT1 encoding arginine methyltransferase, GLT1 encoding glutamate synthase, and VAST1 encoding the VAD1 Analog of StAR-related lipid transfer domain-containing protein, have been reported to be involved in autophagy as well as fungal development and pathogenicity [68,69,70,71]. Despite several well-elucidated components that are involved in autophagy and biological processes in *M. oryzae*, the exact mechanisms of how these components contribute to autophagy and affect pathogenicity are not clear.

### 3.3. Effector-Related Genes

During early infection, fungal pathogens secrete effectors to suppress host immune responses and promote invasion and colonization [20,72]. As effectors evolve stochastically, their biochemical functions can be unpredictable. Many effectors in *M. oryzae* were identified through secreted protein predictions from genome sequences or structural similarities with known effectors from other pathogens. Many avirulence genes, including *Avr-Pita*, *AvrPiz-t*, *Avr-Pia*, *PWL1*, and *PWL2*, have been characterized [73,74,75,76,77]. In addition, four biotrophy-associated secreted proteins (BAS1-BAS4) were identified through transcriptomic analysis [20]. They are invasive hyphae (IH)-specific proteins secreted into host cells with different host compatibilities. However, their mutants for functional analysis have not been obtained [20]. In this section, the genes encoding the non-avirulence secreted proteins or regulators affecting effectors secretion, which contribute to fungal development and pathogenicity, are discussed (Supplementary Table S1c).

Several cytoplasmic effectors that accumulate in the biotrophic interfacial complex (BIC) before being transferred into plant cells have been confirmed by mutant analysis. A glycine-rich secretion protein, Required-for-Focal-BIC-Formation 1 (RBF1), contains a secretion signal that is essential for its accumulation in the BIC before being delivered into the rice cell [78]. It functions in IH differentiation and fungal pathogenicity by engaging in normal BIC formation [78]. Moreover, two nuclear effectors, Host Transcription Reprogramming 1 and 2 (HTR1 and HTR2), are secreted into the cytoplasm of rice cells via the BIC. They serve as transcriptional repressors to reprogram immunity-related genes in host rice [79]. *HTR1* and *HTR2* deletion mutants showed significant defects in fungal pathogenicity [79].

By using a large-scale putative secreted protein genes disruption analysis, MC69 was identified to be essential for IH development, appressoria penetration, and fungal pathogenicity [80]. Unlike the effectors mentioned before, MC69 is secreted and localized to the BIC but cannot be translocated into the plant host cell [80]. Meanwhile, the deletion mutants of the endoplasmic reticulum (ER) chaperone LHS1, *lhs1*, also showed compromised pathogenicity and impaired asexual development [81]. LHS1 is essential for the function and secretion of the avirulence effector AVR-Pita [81]. Two small secreted proteins (MPG1 and MHP1) were identified to be required for the formation of hydrophobin, the small hydrophobic proteins that can be commonly found in filamentous fungi [82,83,84]. They also contribute to fungal development and pathogenicity [82,83]. In contrast, another small secretory protein belonging to the snodprot1 family, MSP1, only plays critical roles in pathogenicity, not in development [85]. Furthermore, the secreted protein-encoding gene *HRIP1* (HR-inducing protein elicitor) has a high level of expression in the fungal penetration and colonization stages, and its deletion mutants showed normal growth and asexual development but exhibited attenuated virulence on rice, suggesting its importance in fungal pathogenicity [86]. In addition, a microarray analysis uncovered two small secreted proteins, Hypothetical Effector Gene13 (HEG13) and HEG16, which contribute to fungal virulence [87]. *HEG13* has a late expression profile that can suppress plant cell death while *HEG16* expresses early and functions in promoting the invasion of epidermal cells and mesophyll colonization [87].

During infection, fungi can also generate pathogen-associated molecular patterns (PAMPs) to facilitate colonization. Chitin is one of the best characterized PAMPs that is released from the fungal cell wall to avoid plant recognition. Chitin oligomers can be deactivated through binding, degradation, or deacetylation [88]. Chitinase 1 (CHIA1), also a secreted protein, can not only bind chitin to suppress plant immune responses, but it can also be recognized by the tetratricopeptide-repeat (TPR) protein in the rice apoplast to trigger PTI [89]. The deletion of *CHIA1* results in delayed fungal development, including conidia germination and appressoria development, as well as decreased fungal virulence [89]. SLP1, the secreted LysM Protein1, is an effector protein secreted into the interface between the fungal cell wall and the plant plasma membrane. It can bind chitin and inhibit the PAMP-induced plant immune responses [90]. The deletion of *SLP1* reduces fungal virulence [90]. Similarly, the auxiliary activity protein AA91, also a chitin-binding protein, is secreted into the apoplast and can suppress the plant immune response as well [91]. *aa91* mutants exhibited abnormal appressoria development and compromised pathogenicity [91]. Moreover, chitin deacetylase CDA7 is an apoplastic effector inhibiting plant immune responses [92]. The *cda7* mutants showed normal fungal morphology and conidia development but reduced appressorium turgor pressure and attenuated virulence, indicating its importance for the fungal full virulence [92].

Several genes that regulate effector secretion also impact fungal development and pathogenicity. Chen et al. (2014) [93] identified an α-1,3-mannosyltransferase, the Asparagine-linked glycosylation3 (ALG3), which mediates the N-glycosylation of the effector SLP1 and influences the SLP1 chitin-binding activity. Mutants with *ALG3* deletion showed delayed infection hyphae development and compromised virulence in rice [93]. The β subunit of the Sec61 complex (SEC61β), the protein-conducting channel for transport, affects the apoplastic effectors SLP1 and BAS4, especially their localizations [94]. It also plays an important role in fungal pathogenicity and development, such as conidiogenesis, cell wall integrity, and appressoria development [94]. Additionally, the syntaxin protein SYN8 and the verprolin protein VRP1 regulate the secretion of *Avr-Pita*- and *Avr-Pia*-encoding effectors, respectively [95,96]. Both *syn8* and *vrp1* mutants showed impaired virulence and abnormal asexual development [95,96]. As secretion is a general cellular process, it is not surprising that mutating the proteins contributing to secretion yields pleiotropic effects.

### 3.4. Signaling Pathways in M. oryzae

**Figure 3:**
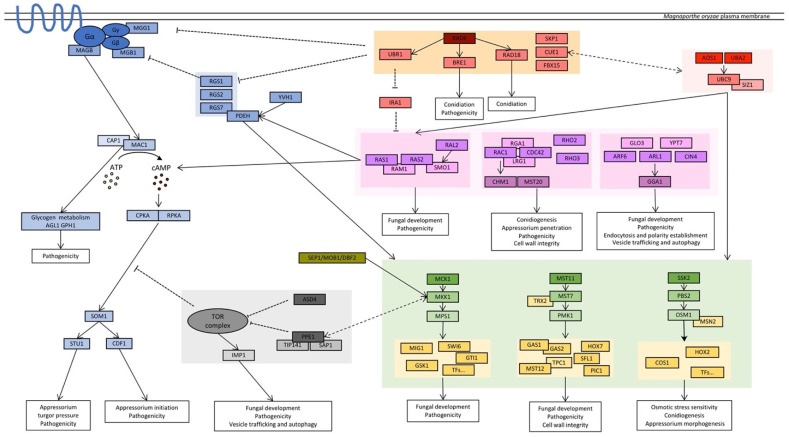
Important signaling pathways in *M. oryzae* that are discussed in this review. G proteins and cAMP/PKA signaling pathways are colored blue. MAPK cascades are colored green. Monomeric GTPase modules (Ras superfamily) are colored pink. Target of Rapamycin (TOR) signaling pathway is colored grey. Ubiquitination pathways are colored orange.

In this section, components of the signaling pathways are discussed (Supplementary Table S1d). These are master contributors to *M. oryzae* biology that regulate a broad range of downstream factors. Therefore, it is not surprising that their disruption dramatically impacts fungal development and pathogenicity. Here, a conceptual model is included, summarizing the major signaling pathways discussed in the following section (Figure 3).

#### 3.4.1. Heterotrimeric G Protein Subunits and Regulatory Proteins

Heterotrimeric guanine nucleotide-binding proteins (G proteins) are molecular switches in signal transduction, transducing signals from cell surface receptors to various intracellular downstream components [97]. In *M. oryzae*, three G protein α subunits (MAGA, MAGB, and MAGC), two β subunits (MGB1 and MGB2), and one γ subunit (MGG1) have been identified [22,98,99,100]. Among them, MAGB, MGB1, and MGG1 are the most well studied through mutant analysis, which are involved in the signaling pathways that regulate fungal vegetative growth, appressoria development, and pathogenicity [98,99,100].

In addition to the G protein subunits, regulators of G-protein signaling (RGS) have also been studied. RGS proteins function as GTPase-accelerating proteins (GAPs), negatively regulating G proteins by turning off the G protein signaling pathways [101,102]. In *M. oryzae*, eight RGS and RGS-like proteins have been characterized. RGS1 was the first identified negative regulator of Gα subunits in *M. oryzae*, which plays important roles in fungal development and pathogenicity [103,104]. It also regulates the expression of effector-encoding genes, contributing to the infection process [105]. Further analysis revealed that RGS2 and RGS6 only contribute to fungal development while RGS3, RGS4, and RGS7 are necessary for both fungal development and pathogenicity [104]. Specifically, RGS2 acts upstream of MAGB for conidiation regulation and RGS7 interacts with MAGB to regulate pathogenicity [104]. In addition, an RGS7-interacting protein, MIP11, is required for fungal development and pathogenicity [106]. It also interacts with PDEH, an important component of the cAMP pathway that will be discussed later [106].

#### 3.4.2. Components of cAMP Pathway

Cyclic AMP (cAMP) can be used by G proteins as a secondary messenger for signal transduction. Lee and Dean (1993) [107] found that exogenous cAMP can induce appressoria formation, indicating the importance of the cAMP-dependent signaling pathway in recognizing surfaces and forming the infection structure [107]. The gene encoding the catalytic subunit of cAMP-dependent protein kinase A, *CPKA*, plays a critical role in appressoria penetration for fungal pathogenicity [108]. Mutants with *CPKA* deletion showed attenuated virulence, but they exhibited normal appressoria formation and invasive hyphal growth in plants that can still infect wounded plants [108]. The regulatory subunit of cAMP-dependent protein kinase A, RPKA, is also essential for fungal development and pathogenicity [109]. CPKA seems to be mainly localized in cytosolic vesicles while RPKA shows a nuclear–cytoplasmic distribution pattern, and this cytoplasmic localization is governed by CPKA [109]. Notably, their interactions and localization can be affected by the exogenous addition of cAMP [109].

Two cAMP phosphodiesterases in *M. oryzae*, PDEL (low affinity) and PDEH (high affinity), were found to engage in intracellular cAMP signaling. *PDEH* deletion mutants showed defects in fungal development and attenuated virulence, suggesting that *PDEH* is a key regulator of cAMP signaling [110]. On the other hand, *PDEL* only plays a minor role in cAMP signaling regulation and may predominantly function in the absence of *PDEH* [110]. In addition, the protein phosphatase YVH1 acts upstream of PDEH to regulate the cAMP signaling pathway [111]. *yvh1* mutants exhibited defects in asexual development, pathogenicity, cell wall integrity, and osmotic stress sensitivity [111].

MAC1 encodes adenylate cyclase, which catalyzes the production of cAMP from ATP [112]. *mac1* mutants exhibited defects in fungal development, including conidiation and conidial germination, along with disabilities in appressoria formation and attenuated virulence [112]. Meanwhile, a MAC1-interacting protein CAP1, also known as the adenylate cyclase-associated protein, plays an important role in MAC1 activation [113]. The deletion of *CAP1* causes a reduced intracellular cAMP level, indicating its involvement in the cAMP pathway [113]. The defects shown in *cap1* mutants, including reduced fungal vegetative growth and conidiation, abnormal appressoria formation, and significantly impaired virulence, can be partially rescued by exogenous cAMP [113].

Several downstream targets regulated by the cAMP/PKA signaling pathway have been functionally studied through target gene deletion analysis. Upon cAMP activation, SOM1 can further activate the transcription factors STU1 and CDTF1 to regulate appressorium turgor and appressorium initiation, respectively [114,115]. Corresponding single-gene deletion mutants showed defects in virulence, indicating that these downstream components of the cAMP/PKA pathway are indispensable in infection-related morphogenesis and pathogenicity. In addition, the cAMP/PKA signaling pathway plays an essential role in glycogen metabolism. Amyloglucosidase (AGL1) and glycogen phosphorylase (GPH1) can inhibit glycogen mobilization during appressoria development and affect fungal virulence [116].

#### 3.4.3. Mitogen-Activated Protein Kinase (MAPK) Cascade

The mitogen-activated protein (MAP) kinase (MAPK) PMK1 acts downstream of the cAMP signaling pathway [117]. *pmk1* mutants exhibited abnormal fungal development and a reduced ability to cause disease in rice and barley [117]. Another two MAP kinase genes, *MPS1* and *OSM1*, have also been studied. *mps1* mutants showed defects in sporulation, fertility, cell wall integrity, and appressoria penetration, resulting in a nonpathogenic phenotype [118]. Unlike *MPS1*, *OSM1* does not regulate appressorium turgor pressure and fungal pathogenicity; it participates in osmotic stress sensitivity and appressorium morphogenesis [119]. However, MSN2, an OSM1-interacting protein containing the C2H2 zinc-finger DNA-binding domain, is required for cell wall integrity and stress responses and contributes to asexual development and fungal pathogenicity [120].

A MAPK kinase cascade is formed by MAPKK kinase (MAPKKK), MAPK kinase (MAPKK), and MAPK. Several MAPKKK-MAPKK-MAPK cascade components have been studied. *MST11* and *MST7* genes encode MAPKKK and MAPKK, respectively, to activate PMK1 [121]. These two genes are necessary for fungal development and pathogenicity, indicating the importance of the MST11-MST7-PMK1 cascade in *M. oryzae* biology [121]. Furthermore, thioredoxin TRX2 interacts with MST7 and regulates PMK1 activation [122]. *trx2* mutants are defective in fungal asexual development and virulence [122].

Furthermore, MCK1 and MKK1 serve as the MPKKK and MAPKK for MPS1 activation, respectively [123,124]. They play essential roles in fungal growth, asexual development, and pathogenicity, as well as the maintenance of cell wall integrity [123,124]. In addition, Yin et al. (2016) [124] found that PDEH acts upstream of this MCK1-MKK1-MPS1 cascade, suggesting an interaction between cAMP signaling and the MAPK cascade [124].

The p21-activated kinases (PAKs), MST20 and CHM1, are homologs of PMK1 in *Saccharomyces cerevisiae* [125]. *MST20* deletion results in reduced aerial hyphae and conidiation but normal appressoria formation and penetration, which are significantly impaired in *CHM1* deletion mutants and lead to a nonpathogenic phenotype [125]. MST20 and CHM1 cannot individually activate the PMK1 MAPK pathway and likely function redundantly in *M. oryzae* [125]. Moreover, SEP1, a component of the Mitotic Exit Network (MEN), functions upstream of MKK1 through phosphorylation and contributes to fungal asexual development, pathogenicity, and cell wall integrity [126]. Other MEN components, including DBF2 and MOB1, are also essential for these processes, indicating a crosstalk between the MEN and MKK1 pathways [126].

Many downstream targets of MAPKs have been functionally studied. For example, the homeobox transcription factor, HOX7, is regulated by the PMK1 MAPK cascade and is involved in appressoria development [127]. MST12, a Cys2-His2 (C2H2) zinc-finger protein, functions downstream of PMK1 to regulate fungal pathogenicity [128]. An MST12-interacting transcription regulator, TPC1, modulates the MST12 DNA-binding affinity [129]. TPC1 plays a critical role in fungal asexual development and pathogenicity, and its nuclear localization is dependent on the PMK1 pathway regulation, suggesting its involvement in the PMK1 MAPK cascade [129]. Meanwhile, the transcription factor SFL1, containing MAPK docking and phosphorylation sites, also interacts with PMK1 and plays critical roles in fungal conidiation and virulence [130]. Furthermore, single-deletion mutants of GAS1 and GAS2, two small proteins regulated by PMK1, show normal growth and asexual development but are unable to penetrate plant hosts to cause disease [131]. Another target of the PMK1 MAPK cascade, PIC5, was identified through a PMK1-interaction screen and was found to be critical in appressoria differentiation and fungal pathogenesis [132].

Among the downstream components of the MPS1 MAPK cascade, the MADS-box transcription factor MIG1 is essential for fungal infection and pathogenicity but does not impact vegetative hyphal growth and appressoria formation [133]. SWI6, an APSES (Asm1, Phd1, Sok1, Efg1, and StuA) family transcription factor, also functions downstream of the MPS1 MAPK cascade. The deletion of *SWI6* showed defective fungal development, such as slow hyphal growth and the abnormal formation of conidia and appressoria [134]. *swi6* mutants exhibited reduced turgor pressure, which resulted in compromised virulence and defects in the cell wall integrity [134]. Moreover, the glycogen synthase kinase GSK1 is also regulated by MPS1 and is vital for fungal vegetative growth, conidiation, and pathogenicity mediated by appressoria development [135]. Additionally, the WOR1/GTI1 transcription factor GTI1 can be indirectly regulated by MPS1 and contributes to fungal asexual development, cell wall integrity, and pathogenicity [136].

#### 3.4.4. Monomeric GTPase Modules (Ras Superfamily)

The Ras superfamily of small guanosine triphosphatases (GTPases) serves as molecular switches for signal transduction [137,138,139]. They can be classified into five subfamilies, including Ras (Ras sarcoma), Rho (Ras homologous), Rab (Ras-like proteins in brain), Ran (Ras-like nuclear), and Arf (ADP-ribosylation factor) proteins [138].

Similar to many other eukaryotic species such as *Schizosaccharomyces pombe*, *M. oryzae* contains two Ras genes, *RAS1* and *RAS2* [137,138,139]. Mutants with *RAS1* deletion showed slight defects in conidiation, while *RAS2* deletion mutants were lethal, indicating a role of RAS2 in *M. oryzae* [139]. Through generating the dominant active allele of *RAS2*, the *RAS2^G18V^* transformants showed abnormal appressoria formation and a nonpathogenic phenotype, further supporting the importance of RAS2 [139]. RAS2 functions upstream of both the cAMP/PKA and PMK1 MAPK pathways [139]. Meanwhile, a GTPase-activating protein interacting with RAS2, SMO1, is involved in RAS regulation [140]. Mutants with *SMO1* deletion exhibited abnormal development, such as nonadherent conidia, smaller appressoria, and attenuated virulence [140]. Moreover, the farnesyltransferase β-subunit RAM1 interacts with both RAS1 and RAS2 and regulates their localization to the plasma membrane [141]. *RAM1* deletion mutants showed impaired conidia and appressoria formations and compromised virulence [141]. However, the addition of exogenous cAMP could restore the defective appressoria formation, indicating that RAM1 functions upstream of the cAMP signaling pathway. In addition, the function of another Ras-like protein, RAL2, in fungal development and pathogenicity has also been revealed through homologous analysis with *S. pombe* [142]. Essential genes in the cAMP/PKA and PMK1 MAPK pathways, such as *PDEH* and *SMO1*, exhibited decreased expression levels in *ral2* mutants, indicating the importance of RAL2 in the cAMP/PKA and PMK1 MAPK pathways [142].

Several members of the Rho family GTPase have been studied in *M. oryzae*. For example, RAC1 plays important roles in fungal conidiogenesis and pathogenicity [143]. It activates the kinase activity of the downstream target CHM1 and subsequently regulates the conidiogenesis [143]. The deletion of gene encoding another member of the Rho family GTPase, *CDC42*, results in defective conidiation, penetration, and virulence [144]. Other Rho family proteins, RHO2 and RHO3, are required for appressoria penetration and fungal pathogenicity [145,146]. *rho3* mutants have shown reduced intracellular cAMP levels [146]. However, in the presence of exogenous cAMP, defects in *rho3* mutants cannot be rescued, while the abnormal appressoria development of *rho2* mutants can be recovered [145,146]. In addition, eight Rho GTPase-activating proteins (Rho GAPs) containing the conserved RhoGAP domain have also been functionally characterized. Interactions between Rho GTPases and these Rho GAPs were detected. Among them, LRG1 and RGA1 interact with RAC1 and CDC42, indicating their roles in the RAC1 and CDC42 pathways [147]. LRG1 and RGA1 are involved in conidiogenesis and appressoria formation [147]. However, only LRG1 is required for fungal virulence and cell wall integrity [147].

Seven Arf small GTPase family members have also been identified. ARF6 is involved in endocytosis and polarity establishment and is vital for fungal asexual development [148]. For the other ARFs, ARL1 and CIN4 are essential for appressoria penetration and infection growth, contributing to fungal pathogenicity [149]. They also function in vesicle trafficking, and CIN4 is involved in BIC formation [149]. The sole adaptor protein of these ARFs, GGA1, also impacts fungal development and pathogenicity [149]. Moreover, GLO3, an ArfGAP protein, is essential for both asexual and sexual development and virulence [150]. The deletion of *GLO3* causes defective endocytosis and responsiveness to endoplasmic reticulum (ER) stress [150]. In addition, Rab GTPase YPT7 is required for asexual development and pathogenicity while being essential for vacuole fusion and autophagy [151]. Interestingly, YPT7 helps to recruit VPS35, the cargo-recognition complex, to the endosome, indicating its indispensable role in autophagy [152].

#### 3.4.5. Target of Rapamycin (TOR) Signaling Pathway

The cAMP/PKA and MAPK pathways are positive regulatory pathways for appressoria formation and development, whereas the Target of Rapamycin (TOR) pathway negatively controls these processes. It acts downstream of cAMP/PKA to inhibit appressoria formation [153]. ASD4, the GATA transcription factor, acts upstream of TOR signaling. ASD4 regulates the expression of genes involved in nitrogen assimilation and glutamine synthesis, such as *GLN1*, and importantly inactivates TOR signaling [153]. In *asd4* mutants, the TOR signaling is activated, resulting in appressoria defects [153].

PPE1, a serine/threonine protein phosphatase, and its partner protein SAP1, regulate the TOR pathway in a negative manner [154]. They are required for normal fungal development, pathogenicity, and cell wall integrity [154]. PPE1 also interacts with MKK1, indicating a complex regulatory network in *M. oryzae* [154]. Moreover, another PPE1-interacting protein, TIP41, is also involved in the TOR pathway [155]. Mutants with *TIP41* deletion were defective in fungal development and virulence, as well as cell wall integrity and autophagy [155]. Interestingly, TIP41 affects the appressorium turgor pressure rather than appressoria formation and is also involved in rapamycin sensitivity [155]. SKP1/BTB/POZ domain-containing protein WHI2 and its interacting phosphatase PSR1 also negatively regulate the TOR pathway [156]. *WHI2* and *PSR1* single-deletion mutants all showed defects in appressoria development and fungal pathogenicity [156]. In addition, IMP1 acts as a downstream target of the TOR signaling pathway [157]. *imp1* mutants exhibited compromised appressoria development and a nonpathogenic phenotype [157]. IMP1 also localizes to vacuoles and heavily promotes vesicle trafficking and autophagy, suggesting a branch signaling of TOR-IMP1-autophagy [157].

#### 3.4.6. Ubiquitination Cascade

The ubiquitin system is a proteolysis pathway conserved in eukaryotes [158]. The ubiquitination of target proteins requires a series of enzymatic reactions. Ubiquitin is first activated by the activating enzyme (E1) and then transferred to the conjugating enzyme (E2). E2 and the substrate protein bind through the ubiquitin ligase (E3). The polyubiquitin chain is generated from subsequent ubiquitin conjugation, which is then recognized by the 26S proteasome for degradation [158]. The exogenous application of the proteasome inhibitor Bortezomib can affect conidia germination, appressoria formation, and the pathogenicity of *M. oryzae* [159]. In addition, the polyubiquitin (*MGG_01282*) is involved in asexual and sexual development and fungal virulence, supporting the importance of ubiquitination in *M. oryzae* biology [159].

The E2 RAD6 and its downstream E3s (BRE1, UBR1, and RAD18) have been functionally characterized [160]. The deletion of *RAD6* results in multiple defects in fungal development and pathogenicity, such as slow vegetative growth, reduced conidia production, and appressoria formation [160]. Further analyses of the downstream E3 ligases revealed that the RAD6-UBR1 cascade plays critical roles in conidia germination and appressoria development [160]. Essential components of the cAMP/PKA, Ras, and PMK1 MAPK signaling pathways, including Gα subunits, RGS1, and the Ras antagonist IRA1, are substrates of the RAD6-UBR1 cascade [160]. In addition, the RAD6-BRE1 cascade regulates histone H3K4 methylation and contributes to fungal conidiation and pathogenicity [160]. Due to the weak interaction between RAD6 and RAD18, the *rad18* mutant showed a normal phenotype [160].

Several studies have illustrated the importance of the ubiquitin system in *M. oryzae* development and pathogenicity. A key component of the SCF (Skp1/Cullin1/F-box) E3 ubiquitin ligase complex, SKP1, is required for fungal development and pathogenicity [161]. The *skp1* RNAi-silenced knockdown transformants showed defects in germination, sporulation, and appressoria formation, exhibiting a nonpathogenic phenotype [161]. Moreover, GRR1, an adaptor to E3 ubiquitin ligase containing the F-box, also contributes to developmental processes, such as conidiogenesis, appressoria formation, turgor pressure generation, cell wall integrity, and virulence [162]. In addition, the ubiquitin system component cue protein (CUE1) and the ubiquitination-associated F-box protein (FBX15) are also involved in fungal conidiation and pathogenicity [163]. Particularly, CUE1, a key factor of the endoplasmic-reticulum-associated degradation (ERAD) complexes, is essential for the ER stress response and the accumulation of cytoplasmic effectors in the BIC [163].

Ubiquitins are recycled by deubiquitinating enzymes (DUBs) that disassemble ubiquitin conjugates into monomeric ubiquitin [164]. Two DUBs, UBP4 and UBP8, have been identified and functionally characterized. Single-deletion mutants of *UBP4* and *UBP8* both exhibited defects in fungal vegetative growth, asexual development, and pathogenicity [165,166]. UBP8 also regulates the carbon catabolite repression in *M. oryzae* [166].

Sumoylation is a post-translational modification similar to ubiquitination, where the small ubiquitin-like modifier (SUMO) attaches to target proteins. The association between ubiquitination and sumoylation has been characterized in the *cue1* and *fbx15* single-deletion mutants, where the reduced sumoylation levels can be observed in these ubiquitination-related deletion mutants [163]. The SUMO pathway in *M. oryzae* has been studied, including the small ubiquitin-like modifier SMT3, E1-activating enzymes AOS1 and UBA2, E2 conjugating ligase UBC9, and E3 ligase SIZ1 [167]. By analyzing these single-gene deletion mutants, the SUMO pathway has been uncovered to play essential roles in multiple processes, including fungal vegetative growth, conidia development, and pathogenicity [167]. Moreover, the SUMO pathway is involved in cellular responses to different stresses, such as osmotic and oxidative stresses [167]. In addition, many candidate downstream targets of SUMO have been identified via affinity purification, GO annotation, and KEGG pathway analysis. Many proteins associated with effector secretion, cell wall integrity, appressoria development, and fungal virulence can be SUMO modified [167]. Some small GTPases (e.g., RHO1 and RAS2) and protein kinases (e.g., PMK1, MPS1, and OSM1) are also putative SUMO proteins [167]. However, more detailed analyses using genetic and molecular methods are needed to define the functions of these substrates and how sumoylation affects their activities.

### 3.5. Multifunctional Genes Involved in Different Aspects of M. oryzae Biology

In this section, master contributors that have profound effects, including different types of transcription factors and kinases and phosphatases, are discussed (Supplementary Table S1e). Interferences of these key components yield pleiotropic defects in both fungal development and pathogenicity.

#### 3.5.1. Transcription Factors

Transcription factors play essential roles in controlling gene expression. The Zn2Cys6 zinc-finger family is the largest family of transcription factors in *M. oryzae* [168]. They have been systematically analyzed, and many of them are involved in both fungal development and pathogenicity [169,170,171]. For example, GPF1 (Growth and Pathogenicity regulatory Factor 1) and CCA1(Conidiation, Conidial germination, and Appressorium formation required transcription factor 1) are required for appressoria penetration, while COD1 and COD2 (COnidia Development 1 and2) control conidia-related genes and are essential for conidiogenesis and pathogenicity [169,170]. LEU3, another leucine-associated Zn2Cys6 TF, acts upstream of other TFs in the same family, including LEU1, LEU2, and LEU4 [171]. These proteins play important roles in the leucine biosynthesis pathway, as well as in fungal development and virulence [171].

The Cys2-His2 (C2H2) zinc-finger protein family also contains a large number of TFs [168]. Most of the C2H2 TFs are involved in diverse aspects of *M. oryzae* biology. For example, CON7 (CONidium morphology) is the first identified C2H2 TF that regulates a large set of genes and contributes to both fungal development and pathogenicity [172]. CRZ1 (calcineurin-responsive transcription factor 1) acts downstream of calcium-dependent signaling and is critical for fungal cell wall integrity and pathogenicity [173]. CREA, a carbon catabolite repressor, affects the expression of many carbon-metabolizing and virulence-related genes [174]. The deletion of *CREA* results in multiple fungal defects, including slow growth, impaired asexual development, and compromised pathogenicity [174]. However, some C2H2 TFs, such as CONx4; CONx6 to CONx11; GCF4 and GCF7 (Growth and Conidiation regulatory Factors); ZAP1; and ZFP2, 3, 9, 14, and 15, only contribute to fungal development [175]. Others, such as ZFP1, 6, 8, and 11, play important roles solely in fungal pathogenicity [175].

Basic leucine zipper (bZIP) transcriptional factors contain a basic DNA-binding region and a leucine-zipper region, which are highly conserved in eukaryotes. The full set of bZIP TFs has been identified through genome sequencing and BLAST analysis. Several of them play significant roles in fungal development and virulence, including BZIP4, 7, 10, 11, 13, 14, and 22; ATF1 (homologous to bZIP TF ATF/CREB from yeasts to mammals); AP1 (homolog of the bZIP TF AP1); METR; and HAC1 [176,177,178,179]. In particular, AP1 and ATF1 are essential for the oxidative stress response, and METR is involved in methionine biosynthesis [176,177,179]. Moreover, YCP4, a target of AP1, is involved in controlling fungal growth, conidiogenesis, appressoria turgor pressure, cell wall integrity, stress responses, and pathogenicity [180].

Homeobox transcription factor genes (*HOX*) contain highly conserved sequences coding for the homeodomain, which is the DNA-binding motif commonly found in developmental regulators. They are essential in fungal growth and differentiation [181,182]. In *M. oryzae*, eight *HOX* genes (*HOX1* to *HOX8*) were identified that played divergent roles in fungal development, including appressoria formation and conidiation [182]. Kim et al. (2009) [182] found that *HOX1* and *HOX6* were involved in hyphal growth, while HOX4 affects conidia size. The *HOX2* deletion mutants exhibited no conidia formation but had similar virulence to WT on rice leaves due to typical mycelium development [182]. *hox7* mutants showed no appressoria formation and a nonpathogenic phenotype when the mutated conidial suspension was sprayed onto rice seedlings [182]. However, the infiltration assay of the *hox7* conidial suspension showed a disease lesion on rice leaves, indicating the role of *HOX7* in appressoria penetration [182]. Among these HOX genes, *HOX3* and *HOX5* did not affect either fungal development or pathogenicity [182]. On the other hand, HOX8, also identified as MST12, interacted with a MAP kinase, which is involved in the fungal pathogenicity [128,182].

Three members of the forkhead-box (FOX) TF gene family, *FKH1*, *HCM1*, and *FOX1*, have been functionally characterized. FKH1 is essential for both fungal development and pathogenicity, while HCM1 only contributes to vegetative growth and conidial germination [183].

Other TFs in *M. oryzae* have also been studied. For instance, *CRF1* encodes a basic helix–loop–helix (bHLH) TF and is essential for fungal virulence [184,185]. CRF1 regulates numerous genes related to carbohydrate and lipid metabolism and thus contributes to phosphorylation during appressoria formation and regulating glycerol metabolism [184,185]. It is also involved in fungal growth and asexual development [184,185]. The pH-regulatory transcription factor PACC is essential for fungal biotrophic growth, asexual development, and pathogenicity [186,187]. Interestingly, it is also involved in fungal alkalinization, and its expression increases with an increasing pH [187]. In addition, *TRA1* (Transcription factor 1) is a TF-encoding gene whose accumulation is regulated by the TF CON7 [188]. TRA1 plays important roles in spores’ adhesion and germination and virulence [188]. TRA1-dependent genes, including *TDG4* and another TF-encoding gene *TDG2*, also contribute to fungal virulence [188].

#### 3.5.2. Kinases and Phosphatases

Protein phosphorylation is a major post-translational modification that is critical in intracellular regulation. This process is revisable in that the targeted proteins can be phosphorylated by kinases at specific sites, and specific phosphatases can help to remove these modifications [189]. In addition to the MAPK pathways, several protein kinases in *M. oryzae* that are essential for fungal development and pathogenicity have been studied.

Subunits of a constitutively active serine/threonine kinase (CK2), CKb1 and CKb2, are required for fungal growth and virulence [190]. CK2 is necessary for a large ring structure formation in the appressorium, which is important for appressoria penetration [190]. The actin-regulating protein ARK1 (Actin-Regulating Kinase 1) is another serine/threonine kinase that is vital for fungal pathogenicity [191]. Mutants with *ARK1* deletion showed abnormal appressorium turgor pressure [191]. The *ark1* mutants were also defective in endocytosis and not sensitive to exogenous oxidative stress [191]. Meanwhile, the related ARK1-interacting actin-binding protein, ABP1, was found to affect the actin cytoskeleton and contribute to fungal growth and pathogenicity [192]. Moreover, the dual-specificity tyrosine-regulated protein kinase YAK1 (orthologous to YAK1 in *S. cerevisiae*) is involved in fungal development and pathogenicity [193]. *YAK1*-deletion mutants exhibited defects in glycogen and lipid metabolism, resulting in decreased turgor pressure and abnormal appressoria penetration [193]. In addition, the cyclin-dependent kinase subunit CKS1 and the atypical guanylate kinase GUK2 are both critical in appressoria development and fungal infection [194,195].

The SNF1/AMP-activated protein kinase (AMPK) family is conserved in eukaryotes to balance the cellular energy ATP [196]. SNF1 is a catalytic subunit of the SNF1/AMPK pathway, and it maintains peroxisomes and lipid metabolism and is essential for sporulation, vegetative growth, and virulence [197,198]. In *M. oryzae*, the β-subunit SIP2, γ-subunit SNF4, and SNF1-activating kinases SAK1 and TOS3 have been shown to play critical roles in lipid metabolism, fungal development, and virulence [198].

Histidine kinases (HIKs) are sensor proteins for external signal detection and signaling [199]. They form unstable phosphoramidate bonds, which are different from serine/threonine kinase-producing phosphoester bonds [200]. In *M. oryzae*, *SLN1*, which encodes a putative histidine kinase, functions as a turgor sensor and is essential for cell wall integrity [201,202]. Its deletion mutants exhibited an impaired melanin layer, decreased appressorium turgor pressure, and attenuated virulence [201,202]. The histidine kinase PAS1 (period circadian protein, aryl hydrocarbon receptor nuclear translocator protein, and single-minded protein) is also required for mycelia and conidial development, appressoria formation, and pathogenicity [203]. Several HIKs-encoding genes, *HIK1* to *HIK9*, have been identified, and their single-deletion mutants have been functionally characterized. They contribute to conidial development and fungal virulence in varying degrees [199]. HIK5 plays some significant roles as its deletion mutants cannot cause any disease on the plant host and showed defects in appressoria formation and cell wall integrity [199]. Similarly, *HIK8*-deletion mutants were also nonpathogenic [199]. In addition, HIK5 and HIK9 are involved in cell wall integrity and the hypoxia-sensing pathway [199].

PPG1, the serine/threonine-protein phosphatase catalytic subunit (PP2A), is critical for vegetative growth, conidiation, and appressoria penetration in *M. oryzae* [204]. It also regulates the Rho family GTPase, such as CDC42, RHO3, and RAC1, which are key factors for pathogenicity [204]. The phosphatase CDC14 antagonizes cyclin-dependent kinases, which affects mitosis and cytokinesis [205]. The deletion of *CDC14* results in reduced growth and conidiation, an abnormal septation and nuclei distribution in the hyphae, and appressoria formation defects, which ultimately results in compromised pathogenicity [205].

TPS2, the trehalose 6-phosphate phosphatase, is a key component of the trehalose phosphate synthase/trehalose phosphate phosphatase (TPS/TPP) pathway for trehalose biosynthesis [206]. Mutants with *TPS2* deletion showed defects in fungal growth, conidiogenesis, and cell wall integrity [206]. The *tps2* mutants also exhibited abnormal turgor pressure, which resulted in compromised virulence [206]. In addition to TPS2, the TPS complex in *M. oryzae* contains another two subunits, trehalose 6-phosphate synthase (TPS1) and a regulatory subunit (TPS3). TPS1 also plays an essential role in fungal infection and carbon and nitrogen metabolism, while TPS3 is required for TPS1 activation and contributes to fungal pathogenicity [207].

In *M. oryzae*, five lipid phosphate phosphatases (LPP1 to LPP5) have been identified. Among them, only LPP3 and LPP5 are involved in diacylglycerol regulation and are critical for appressoria development and fungal virulence [208]. In addition, the phosphatidate phosphatase PAH1, expressed in multiple stages, contributes to asexual development, heat tolerance, and fungal virulence [209]. The *PAH1* deletion mutants exhibited lipid alteration, which affected the accumulation of phosphatidic acid, suggesting its essential role in lipid metabolism [209].

#### 3.5.3. Peroxisomal- and Mitochondrial-Related Genes

Peroxisomes are single-membrane organelles that participate in several lipid metabolisms [210]. According to their role or localization, peroxisome proteins can be divided into three groups: biogenesis proteins (peroxins), matrix proteins, and membrane proteins [211]. In *M. oryzae*, several peroxisome proteins, including PEX1, PEX5, PEX6, PEX7, PEX13, PEX14, PEX14/17, and PEX19, have been studied. They are important for fungal development and pathogenicity [210,211,212,213,214,215,216,217].

In particular, PEX13 and PEX14 are key components of the peroxisomal docking complex and are essential for peroxisome formation [213]. The peroxisomal fission gene 1 (*PEF1*) also regulates peroxisome formation by affecting peroxisomal fission [218]. On the other hand, PEX5 and PEX7 are the receptors for peroxisomal targeting signal (PTS) 1 and PTS2, respectively, which are essential for recognizing and importing peroxisomal matrix proteins [211,216,219]. PEX6 is also required for peroxisomal integrity and subsequently affects the import of matrix proteins and β-oxidation of fatty acids [210]. In addition, PEX19 is required to maintain the peroxisomal structure while PEX11A plays a critical role in peroxisomal proliferation [212,215].

The generation of acetyl-CoA is one of the most important consequences of fatty acid β-oxidation [220]. PTH2 is a carnitine acetyl-transferase (CAT) that is predominantly present in peroxisomes [220]. The *pth2* mutants lost the CAT activities and showed a compromised lipid metabolism in appressoria and attenuated fungal virulence, indicating the importance of acetyl-CoA in appressoria function and fungal pathogenicity [220]. Meanwhile, the peroxisomal-CoA synthetase PCS60 is also involved in fatty acid metabolism and contributes to the growth of infection hyphae and fungal pathogenicity [221]. The β-oxidation of fatty acids also results in the reoxidation of NADH to NAD+ in peroxisomes. The alanine, glyoxylate aminotransferase 1 (AGT1), is localized to peroxisomes and exhibits an indispensable role in fungal full virulence [222]. The disruption of *AGT1* affects the ratio of ADH/NAD+ in peroxisomes, resulting in defects in lipid mobilization and turgor pressure generation [222]. As a consequence, the *agt1* mutants displayed abnormal appressoria penetration and impaired pathogenicity [222].

In addition to peroxisomes, the β-oxidation of fatty acids also occurs in mitochondria, and many mitochondrial-localized enzymes also play critical roles in *M. oryzae* biology [223]. The short-chain acyl-CoA dehydrogenase 2 (SCAD2) is involved in the first dehydrogenation step of the mitochondrial β-oxidation pathway [224]. The disruption of *SCAD2* leads to an impaired ability to utilize fatty acids [224]. The *scad2* mutants exhibited defects in appressoria development and fungal pathogenicity [224]. Meanwhile, Acyl-CoA dehydrogenases are also involved in the respiratory system, delivering electrons to the ubiquinone pool to synthesize ATP with the help of electron-transferring flavoprotein (ETF) and its dehydrogenases (ETFDH) [225]. In *M. oryzae*, two subunits of the ETF (ETFA and ETFB) and one ETFDH all localize to mitochondria and function downstream of the mitochondrial β-oxidation [226]. Single deletion mutants of these genes showed defects in fungal vegetative growth, conidiation, virulence, and fatty acid metabolism [226]. Another mitochondrial β-oxidation enzyme, Enoyl-CoA hydratase ECH1, is also required for the fungal utilization of fatty acids [227]. The *ech1* mutants had defects in mitochondrial β-oxidation and integrity, conidial germination, abnormal appressoria penetration, and compromised fungal virulence [227].

The 3-methylglutaconyl-CoA hydratase AUH1 contributes to the fusion and fission of mitochondria [228]. Mutants with *AUH1* deletion displayed defects in fungal development and pathogenicity [228]. Another mitochondrial fission protein FIS1 is also crucial for both fungal development and virulence [229]. Moreover, a FIS1-interacting protein DNM1, also known as the dynamin-related protein, is required for asexual development, such as conidiation and turgor pressure generation and pathogenicity [230]. Interestingly, the localization of DNM1 can be observed in both peroxisomes and mitochondria, and it plays a critical role in both peroxisomal and mitochondrial fission [230]. In addition, the isovaleryl-CoA dehydrogenase IVD, another mitochondrial-localized enzyme, is involved in leucine catabolism and contributes to fungal vegetative growth, conidiation, and pathogenicity [231]. The acetoacetyl-CoA acetyltransferase ACAT1 is also mitochondria localized, whereas ACAT2 is localized to the cytoplasm [232]. Although both ACAT1 and ACAT2 are required for the fungal full virulence, only ACAT2 is involved in vegetative growth [232].

#### 3.5.4. Other Important Genes in *M. oryzae* Biology

The turgor pressure produced by appressorium requires the formation of a 1,8-dihydroxynaphthalene (DHN) melanin layer as an impermeable barrier [233]. In *M. oryzae*, three melanin synthesis genes (*ALB1*, *RSY1*, and *BUF1*) have been functionally studied [234]. Besides their important roles in turgor pressure generation, they are also required for fungal virulence [235]. Their mutants showed defects in conidiation, appressoria formation, and stress resistance [235].

Indole-3-acetic acid (IAA) is the most common auxin involved in plant growth, development, and plant–microbe interactions [236]. The IAA/auxin biosynthesis also occurs in fungal pathogens. The indole-3-pyruvic acid (IPA) pathway in *M. oryzae* plays essential roles in the IAA/auxin biosynthesis, as well as fungal development and pathogenicity [237]. The tryptophan aminotransferase (TAM1) and the indole-3-pyruvate decarboxylase (IPD1) are key components of the IPA pathway [237]. The deletion of *TAM1* and *IPD1* leads to reduced IAA production [237]. In addition, *tam1* and *ipd1* mutants exhibited defects in vegetative growth and conidiation and impaired virulence [237].

Transcription and post-transcriptional levels of regulations play key roles in *M. oryzae* biology [238]. RNA interference (RNAi) is a conserved mechanism of transcriptional regulation, where small interfering RNAs (siRNAs) bind to target sequences and silence the gene expression [238]. RNAi pathway components of *M. oryzae*, including the primary Dicer (DCL2), the RNA-dependent RNA polymerase (RDRP1), and Argonaute (AGO3) are required for siRNA biogenesis [239]. The disruption of *DCL2*, *RDRP2*, and *AGO3* results in reduced conidia production [239]. Furthermore, deletion mutants of *RDRP2* and *AGO3* were unable to colonize and infect plant hosts [239]. These results suggest the essential roles of siRNA in fungal development and pathogenicity [239]. Moreover, RNA methylation is one of the regulatory processes of RNA modification, including the reversible and conserved N6-methyladenosine (m6A) RNA methylation [240,241]. The N6-adenosine-methyltransferase (IME4) is required for m6A RNA methylation in *M. oryzae* and is involved in fungal pathogenicity [242]. In addition, two m6A-binding proteins (YTH1 and YTH2) and the mRNA:m6A demethylase (ALKB1) all contribute to fungal virulence, and YTH2 is also important for conidiation [242].

Histone modifications, such as methylation and acetylation, also contribute to the regulation of biological processes at the transcriptional level. In *M. oryzae*, the transcriptional regulation of histone modification dynamics is essential for regulating virulence genes [243]. Several enzymes involved in histone modifications regulate gene expression and contribute to fungal development and pathogenicity. For example, the histone lysine methyltransferase SET1 is required for histone H3 lysine 4 methylation and is involved in numerous gene regulations [244]. The deletion of *SET1* results in defects in vegetative growth and asexual development, including conidiation and appressoria formation [244]. *set1* mutants displayed a compromised ability of plant infection [244]. Moreover, the histone acetyltransferases RTT109 and SAS3 are required for the acetylation of histone H3 lysine 56 (H3K56) and H3K14, respectively [245,246]. The deletion of *RTT109* or *SAS3* results in abnormal asexual development and attenuated virulence [245,246]. Two histone deacetylases, RPD3 and HST4, also contribute to vegetative growth and conidiation [247]. Interestingly, the deletion of *HST4* results in reduced virulence, while the overexpression of *RPD3* leads to the nonpathogenic phenotype, suggesting a negative role of RPD3 in fungal virulence [247]. Furthermore, *TIG1*-, *HOS2*-, *SNT1*-, *SET3*-, and *HST1*-encoding proteins are components of the histone deacetylase (HDAC) transcriptional corepressor complex [248]. Single-deletion mutants of these genes were unable to cause disease on plant hosts and exhibited defects in conidiation [248]. In addition, the histone demethylase JMJ1 is also involved in fungal vegetative growth, appressoria formation, and virulence [249]. Overall, these studies indicate the implication of histone modification in fungal development and pathogenicity.

Many other factors, such as NADPH oxidase-encoding genes (*NOX1* and *NOX2*), O-mannosyltransferases-encoding genes (*PMT2* and *PMT4*), and phospholipase C genes (*PLC1* to *PLC3*) play diverse roles in *M. oryzae* biology [250,251,252,253,254]. Detailed information, including the gene code, mutant name, gene function, and mutant phenotypes can be found in the supplementary table (Supplementary Table S1f).

## 4. Conclusions

Rice blast is one of the worst agricultural diseases in terms of world-wide economic losses. Genetic and genomic analyses of the causal pathogen, *M. oryzae*, have improved our understanding of the disease. Over the past decades, numerous genes involved in the development and pathogenicity of *M. oryzae* have been identified and functionally characterized. Various signaling pathways and virulence factors have been revealed. Evidently, the pathogen uses various strategies to invade the host and overcome the host’s defense responses. These include the formation of invasive structures, such as appressoria and infectious hyphae, and the delivery of diverse effectors.

With more than 10,000 genes encoded in its genome, only about 400 have been fully studied using mutant analysis. This highlights the limited understanding of this important staple cereal pathogen. With the development of target-gene deletion methods, especially the robust and efficient CRISPR technology, genetic studies in *M. oryzae* have been and will continue to be improved. More research is needed to understand the network details involved in its various biological processes. In addition, novel strategies can be applied to confer host resistance to *M. oryzae* based on mutant studies of this pathogen. For example, host-induced gene silencing, a plant engineering technology using RNAi to silence target genes through trans-species RNAi, can be tested and used on some well-studied pathogenicity genes of *M. oryzae*.

Due to the crosstalk of signaling pathways and the complicated network among regulators in *M. oryzae*, identifying specific functions of development or virulence factors can be challenging with mutant analysis. Future developments in cell biology and biochemical tools for targeted analyses will foster a more comprehensive understanding of *M. oryzae* mechanisms. Additionally, collaborations between molecular biologists and breeders will be essential to provide more opportunities for disease management and reduce incidences of rice blast in the field.

## Supplementary Materials

The following supporting information can be downloaded at https://www.mdpi.com/article/10.3390/pathogens12030379/s1. Table S1: Summary of genes from *M. oryzae* that have been studied using mutant analysis. References [255,256,257,258,259,260,261,262,263,264,265,266,267,268,269,270,271,272,273,274,275,276,277,278,279,280,281,282,283,284,285,286,287,288,289,290,291,292,293,294,295,296,297,298,299,300,301,302,303,304,305,306,307,308,309,310,311,312,313,314,315,316,317,318,319,320,321,322,323,324,325,326,327,328,329,330,331,332,333,334,335,336] are cited in the supplementary materials.
