# Supplementary material for: The Devastating Rice Blast Airborne Pathogen Magnaporthe oryzae—A Review on Genes Studied with Mutant Analysis
Source: Pathogens. 2023 Feb 26;12(3):379. doi: 10.3390/pathogens12030379 (PMC10055536; doi:10.3390/pathogens12030379)
Supplement: Supplementary file 1 [file pathogens-12-00379-s001.zip › pathogens-2195325-supplementary.pdf]

Supplementary Table S1: Summary of genes from *M. oryzae* that have been studied using mutant analysis.
(a) Genes mainly related to fungal development.

Gene Code	Fungal Reference Strain	Mutant Name	Gene Full Name/ Encoding Protein	Main Functions	Mutant Type	Mutant Phenotypes				Tested Hosts	Reference
						Hyphal Growth	Conidia Growth/ Development	Appressoria Formation/ Development	Virulence		
MGG_03538	Guy11	*mgwc-1*	white collar-1	a blue light receptor	deletion	+	+	NA	NA		[39]
MGG_03977	M2942 Y34	*cos1*	conidiospore stalk-less1	conidiation	T-DNA insertion deletion	+	+	NA	-	barley and rice leaves rice axils	[40]
MGG_04317	JL0910 (isolated and purified from Oryza sativa cultivar Jijing88)	*zfc3*	C3HC type zinc-finger domain protein	conidia production, mitochondrial ATP-dependent Lon protease (MAP1) interaction	deletion	+	+	NA	-	rice seedlings (cv. JJ88) and barley (cv. Golden Promise)	[41]
MGG_03620	70-15	*st1*	hexose transporter family protein	conidiation and mycelial melanisation	deletion	+	+	-	-	barley cotyledons (cv. Nigrate) and rice seedlings (cv. Shin No. 2)	[42]
MGG_14966	Guy11	*cda1*	chitin deacetylases	chitin deacetylation (hyphae in colony interiors)	deletion	+	-	-	-	rice seedlings (cv. CO39)	[43]
MGG_08774	Guy11	*cda4*	chitin deacetylases	chitin deacetylation (hyphae at colony margins)	deletion	+	-	-	-	rice seedlings (cv. CO39)	[43]
MGG_04118	S6	*fas1*	fatty acid synthase beta subunit dehydratase	lipid biosynthesis, conidiogenesis and appressorium formation	deletion	+	+	+	NA	NA	[44]

MGG_07964	B157	*laea*	global regulator of secondary metabolism	penicillin G biosynthesis	deletion	-	+	-	-	rice seedlings (cv. HR12)	[45]
MGG_14872	Guy11	*capn1*	calpains-related gene	conidiation	deletion	-	+	-	-	rice and barley seedlings	[46]
MGG_03703	P131	*spa2*	spindle pole antigen gene	polar cell growth of vegetative hyphae and conidia	deletion	+	+	-	-	rice (cv. LTH) and barley (cv. C9)	[47]
MGG_05336	Guy11	*mtp1*	type III integral transmembrane protein	conidiation and conidial germination	deletion	NA	+	+	-	rice seedlings	[48]
MGG_01076	P2	*hda1*	class II histone deacetylases HDAC genes	vegetative growth and conidiation	deletion	+	+	-	NA	NA	[49]
	Guy11 (MAT1-1), TH16 (MAT1-1), 2539 (MAT1-2), 70-15 (MAT1-2) and TH3 (MAT1-2)	*mat 1-1*, *mat 1-2*	mating type genes	sexual development - asci and ascospores formation	deletion	-	-	-	-	rice seedlings	[50]

(b) Autophagy-related genes.

Gene code	Fungal Reference Strain	Mutant Name	Gene Full Name/ Encoding Protein	Main Functions	Mutant Type	Mutant Phenotypes				Tested Hosts	Reference
						Hyphal Growth	Conidia Growth/ Development	Appressoria Formation/ Development	Virulence		
MGG_06393	Guy11	*atg1*	serine/threonine protein kinase	appressoria turgor and pathogenesis, initiation of autophagy	deletion	+	+	-	+	barley (H. vulgare cv. ZJ-8) rice (O. sativa cv. CO-39)	[52]
MGG_16734	Guy11	*atg2*	peripheral membrane protein	recycling	deletion	NA	NA	+	+	rice seedling (cultivar CO-39)	[51]
MGG_02959	Guy11	*atg3*	autophagy-related protein	autophagosome formation	deletion	NA	NA	+	+	rice seedling (cultivar CO-39)	[51]
MGG_03580	Guy11	*atg4*	cysteine protease	cleavage of the carboxyl terminus of Atg8, autophagosome formation	deletion	+	+	+	+	rice seedling (cultivar CO-39)	[55]
MGG_09262	Guy11	*atg5*	autophagy-related protein	cell differentiation and pathogenesis, autophagosome formation	deletion	NA	+	+	+	rice seedling (cultivar CO-39)	[58]
MGG_03694	Guy11	*atg6*	autophagy-related protein	nucleation	deletion	NA	+	+	+	rice seedling (cultivar CO-39)	[51]
MGG_07297	Guy11	*atg7*	E1 family of ubiquitin-activating enzyme	autophagosome formation	deletion	NA	NA	+	+	rice seedling (cultivar CO-39)	[51]

MGG_01062	Guy11	*atg8*	autophagy-related protein	autophagosome formation	deletion	+	+	+	+	rice seedling (cultivar CO-39)	[13]
MGG_09559	Guy11	*atg9*	phospholipid scramblase, transmembrane protein	autophagy and cytoplasm to vacuole transport (Cvt) vesicle formation	deletion	+	+	+	+	rice seedling (cultivar CO-39)	[57]
MGG_14737	Guy11	*atg10*	E2-like conjugating enzyme	autophagosome formation	deletion	NA	NA	+	+	rice seedling (cultivar CO-39)	[51]
MGG_04486	Guy11	*atg11*	selective autophagy-related protein	pexophagy	deletion	NA	-	-	-	rice seedling (cultivar CO-39)	[51]
MGG_00598	Guy11	*atg12*	ubiquitin-like modifier	autophagosome formation	deletion	NA	NA	+	+	rice seedling (cultivar CO-39)	[51]
MGG_00454	Guy11	*atg13*	phosphorylated protein	initiation of autophagy	deletion	NA	NA	+	+	rice seedling (cultivar CO-39)	[51]
MGG_03698	Guy11	*atg14*	autophagy-related protein	autophagy and conidial autophagic cell death	deletion	+	+	+	+	rice seedling (cultivar CO-39)	[56]
MGG_12828	Guy11	*atg15*	lipase	recycling	deletion	NA	NA	+	+	rice seedling (cultivar CO-39)	[51]
MGG_05255	Guy11	*atg16*	autophagy-related protein	pre-autophagosomal structure formation	deletion	NA	NA	+	+	rice seedling (cultivar CO-39)	[51]
MGG_07667	Guy11	*atg17*	autophagy-related protein	initiation of autophagy	deletion	NA	NA	+	+	rice seedling (cultivar CO-39)	[51]

MGG_03139	Guy11	*atg18*	phosphatidylinositol 3,5-bisphosphate-binding protein	recycling	deletion	NA	NA	+	+	rice seedling (cultivar CO-39)	[51]
MGG_03638	Guy11	*atg24*	selective autophagy-related protein	sorting nexin	deletion	NA	-	-	-	rice seedling (cultivar CO-39)	[51]
MGG_03459	Guy11	*atg26*	selective autophagy-related protein, UDP-glucose:sterol glucosyltransferase	synthesis of sterol glucoside membrane lipids	deletion	NA	-	-	-	rice seedling (cultivar CO-39)	[51]
MGG_02386	Guy11	*atg27*	selective autophagy-related protein	autophagy	deletion	NA	-	-	-	rice seedling (cultivar CO-39)	[51]
MGG_08061	Guy11	*atg28*	selective autophagy-related protein	degradation of peroxisomes	deletion	NA	-	-	-	rice seedling (cultivar CO-39)	[51]
MGG_02790	Guy11	*atg29*	selective autophagy-related protein	autophagosome formation	deletion	NA	-	-	-	rice seedling (cultivar CO-39)	[51]
MGG_01096	B157	*sga1*	vacuolar glucoamylase (sporulation specific GlucoAmylase)	glycogen autophagy for asexual differentiation	deletion	-	+	NA	-	barley leaf explants	[54]
MGG_03026	Guy11	*nima*	protein kinase	mitosis	deletion	+	+	+	NA	NA	[13]
MGG_09497	Guy11	*opy2*	overproduction-induced pheromone-resistant protein 2	fungal development, pathogenicity, and autophagy	deletion	+	+	+	+	rice seedlings (cv. CO-39)	[59]

MGG_08097	Guy11	*yck1*	casein kinase	development, autophagy, and virulence	deletion	+	+	+	+	rice seedling	[60]
MGG_05379	Guy11	*vps9*	VSP9 domain-containing protein	autophagy and endocytosis, fungal development and pathogenicity	deletion	+	+	+	+	rice seedling (cv. CO39)	[61]
MGG_06375	Guy11	*hat1*	histone acetyltransferase	fungal development and pathogenicity	deletion	+	+	+	+	rice seedling (cultivar CO-39)	[62]
MGG_03677	RFP-ATG8 strain	*gcn5*	histone acetyltransferase	autophagy, conidiation, pathogenicity	deletion	NA	+	NA	+	barley leaf explants	[63]
MGG_05089	Δku70	*vps35*	cargo-recognition complex component	fungal development and pathogenicity, conidial autophagic cell death regulation, biogenesis of autophagosomes	deletion	+	+	+	+	rice seedling (cv.CO-39) and barley (cv. Golden Promise)	[64]
MGG_04830	Δku70	*vps26*	cargo-recognition complex component	fungal conidiation and pathogenicity	deletion	NA	+	NA	+	rice seedling (cv.CO-39) and barley (cv. Golden Promise)	[64]
MGG_02524	Δku70	*vps29*	cargo-recognition complex component	fungal conidiation and pathogenicity	deletion	NA	+	NA	+	rice seedling (cv.CO-39) and barley (cv. Golden Promise)	[64]

MGG_00455	Guy11	*hse1*	component of the endosomal sorting complexes required for transport 0	fungal development, pathogenicity, and autophagy	deletion	+	+	+	+	rice (cv. CO-39) and barley	[65]
MGG_04958	Guy11	*vps27*	component of the endosomal sorting complexes required for transport 0	fungal development, pathogenicity, and autophagy	deletion	+	+	+	+	rice (cv. CO-39) and barley	[65]
MGG_04174	Guy11	*snf7*	core protein of endosomal sorting complex required for transport (ESCRT)-III subcomplex	fungal development and pathogenicity, cell wall integrity, endocytosis, autophagy	deletion	+	+	+	+	rice and barley leaves	[66]
MGG_01765	Guy11	*ist1*	subunit of ESCRT-III	fungal development and pathogenicity, autophagy	deletion	+	+	+	+	rice seedlings (cv. CO-39) and barley	[65]
MGG_09205	Guy11	*hrd1*	ER-associated degradation (ERAD) ubiquitin ligase	fungal development and pathogenicity, lipid metabolism and conidial autophagic cell death	deletion	+	+	+	+	rice (cv. CO-39)	[67]
MGG_06444	Guy11	*der1*	ER-associated degradation (ERAD) ubiquitin ligase	fungal development and pathogenicity, lipid metabolism and conidial autophagic cell death	deletion	+	+	+	+	rice (cv. CO-39)	[67]
MGG_04421	Guy11	*snt2*	DNA-binding domain SaNT deacetylation of H3	autophagy and pathogenicity, regulation of MoATG6, 15, 16, and 22 expressions	deletion	+	+	+	+	rice leaves and rice leaf sheath	[68]

MGG_04584	Guy11	*hmt1*	arginine methyltransferase gene	formation of autophagosomes, fungal development and pathogenicity	deletion	+	+	+	+	rice seedlings (cv CO-39) and barley seedlings	[69]
MGG_07187	P131 and S1528	*glt1*	glutamate synthase	glutamate homeostasis, autophagy, fungal development and pathogenicity	deletion	-	+	+	+	rice seedling (cv. LTH) and barley (cv. E9)	[70]
MGG_11211	Guy11	*vast1*	VAD1 Analog of StAR-related lipid transfer (VASt) domain-containing protein	fungal development and pathogenicity, membrane tension, sterol homeostasis	deletion	+	+	+	+	rice (cv. CO-39)	[71]

(c) Genes encoding effectors.

Gene Code	Fungal Reference Strain	Mutant Name	Gene Full Name/ Encoding Protein	Main Functions	Mutant Type	Mutant Phenotypes				Secretion Signal	Tested Hosts	Reference
						Hyphal Growth	Conidia Growth/ Development	Appressoria Formation/ Development	Virulence			
MGG_10010	Ina86-137	*rbf1*	required-for-focal-BIC-formation 1, glycine-rich secretion protein	biotrophic interfacial complex (BIC) formation and fungal pathogenicity	deletion	-	-	-	+	Yes	rice	[78]
MGG_10276	KJ201	*htr1*	nuclear effector, C2H2 zinc finger protein	fungal pathogenicity	deletion	-	-	-	+	Yes	rice (cv. Nakdong)	[79]
MGG_10280	KJ201	*htr2*	nuclear effector, C2H2 zinc finger protein	fungal pathogenicity	deletion	-	-	-	+	Yes	rice (cv. Nakdong)	[79]
MGG_02848	Ina72	*mc69*	secreted protein, harboring a putative N-terminal secretion signal peptide	appressorial penetration and pathogenicity	deletion	-	-	+	+	Yes	barley cotyledons (cv. Nigrate) and rice seedlings (cv. Shin No. 2 or cv. Sasanishiki)	[80]
MGG_06648	70-15	*lhs1*	ER chaperone, members of the heat shock protein 70 family	effector secretion, fungal development and pathogenicity	deletion	+	+	+	+	Yes	rice (compatible cultivar Nakdongbyeo) plants	[81]
MGG_10315	Guy11	*mpg1*	hydrophobin, small secreted protein	fungal development and pathogenicity, hydrophobin formation	deletion	NA	+	+	+	Yes	rice seedlings (cv. CO39)	[82]

MGG_01173	70-15	*mhp1*	class II hydrophobin, small secreted protein	fungal development and pathogenicity, hydrophobin formation	deletion	+	+	+	+	Yes	rice seedlings (cv. Nakdong)	[83]
MGG_05344	70-15	*msp1*	small secreted protein, snodprot1	fungal virulence	deletion	-	NA	-	+	Yes	rice and barley plants	[84]
MGG_15022	KJ201	*hrip1*	secreted effector	fungal pathogenicity	deletion	-	-	-	+	Yes	rice (cv. Nipponbare)	[86]
MGG_09378	INA72	*heg13*	hypothetical effector gene13	suppress NLP-induced cell death, fungal penetration and pathogenicity	deletion	NA	NA	+	+	Yes	barley primary leaves (cv. Vada)	[87]
MGG_01149	INA72	*heg16*	hypothetical effector gene16	mesophyll colonization, fungal pathogenicity	deletion	NA	NA	NA	+	Yes	barley primary leaves (cv. Vada)	[87]
MGG_08054	Guy11	*chia1*	secreted protein, chitinase	fungal development and pathogenicity	deletion	+	+	+	+	Yes	rice seedling	[89]
MGG_10097	Guy11	*slp1*	secreted LysM protein1	sequesters chitin oligosaccharides, fungal pathogenicity	deletion	-	-	-	+	Yes	rice (cv. CO-39)	[90]
MGG_06069	Guy11	*aa91*	auxiliary activity family 9 protein	appressorium development and virulence	deletion	NA	NA	+	+	Yes	rice (cv. CO39)	[91]
MGG_05023	KJ201	*cda7*	chitin deacetylase	fungal pathogenicity	deletion	-	-	-	+	Yes	rice	[92]

MGG_08010	P131	*alg3*	α-1,3-mannosyltransferase	fungal pathogenicity	deletion	-	-	-	+	No	rice (cv. LTH) seedlings and barley seedlings (cv. E9)	[93]
MGG_03644	Guy11	*sec61β*	beta subunit of Sec61	fungal development and pathogenicity	deletion	+	+	+	+	NA	rice (cv CO-39)	[94]
MGG_06521	Guy11 and JS153	*syn8*	syntaxin protein	fungal development and pathogenicity	deletion	+	+	+	+	No	rice (cv. CO-39) and barley	[95]
MGG_11243	Guy11	*vrp1*	verprolin protein	fungal asexual development and pathogenicity	deletion	+	+	+	+	NA	rice (cv. CO-39)	[96]

(d) Genes involved in different signaling pathways.

Gene code	Fungal Reference Strain	Mutant Name	Gene Full Name/ Encoding Protein	Main Functions	Mutant Type	Mutant Phenotypes				Tested Hosts	Reference
						Hyphal Growth	Conidia Growth/ Development	Appressoria Formation/ Development	Virulence		
**G-protein**											
MGG_01818	70-6 70-15	*maga*	G protein α subunit gene	NA	deletion	-	-	-	-	rice (cv. S-201)	[98]
MGG_00365	70-6 70-15	*magb*	G protein α subunit gene	fungal development and pathogenicity	deletion	+	+	+	+	rice (cv. S-201)	[98]
MGG_04204	70-6 70-15	*magc*	G protein α subunit gene	conidia formation	deletion	-	+	-	-	rice (cv. S-201)	[98]
MGG_05201	Guy11	*mgb1*	G protein β subunit gene	fungal development and pathogenicity	deletion	-	+	+	+	rice (cv. CO39) and barley	[99]
MGG_10193	Guy11	*mgg1*	Gγ subunit gene	fungal development and virulence	T-DNA insertion and deletion	-	+	+	+	rice (cv. CO-39)	[100]
**G protein signaling pathways**											
MGG_14517	Guy11	*rgs1*	regulator of G-protein signaling (RGS) protein	fungal development and pathogenicity	deletion	+	+	+	+	rice leaves (cultivar CO-39)	[103-105]
MGG_03146	Guy11	*rgs2*	regulator of G-protein signaling (RGS) protein	fungal development	deletion	-	+	+	-	rice leaves (cultivar CO-39)	[104]

MGG_03726	Guy11	*rgs3*	regulator of G-protein signaling (RGS) protein	fungal development and pathogenicity	deletion	-	+	+	+	rice leaves (cultivar CO-39)	[104]
MGG_00990	Guy11	*rgs4*	regulator of G-protein signaling (RGS) protein	fungal development and pathogenicity	deletion	+	+	+	+	rice leaves (cultivar CO-39)	[104]
MGG_08735	Guy11	*rgs5*	regulator of G-protein signaling (RGS) protein	-	deletion	-	-	-	-	rice leaves (cultivar CO-39)	[104]
MGG_09618	Guy11	*rgs6*	regulator of G-protein signaling (RGS) protein	fungal development	deletion	+	-	+	-	rice leaves (cultivar CO-39)	[104]
MGG_11693	Guy11	*rgs7*	regulator of G-protein signaling (RGS) protein	fungal development and pathogenicity	deletion	-	-	+	+	rice leaves (cultivar CO-39)	[104]
MGG_13926	Guy11	*rgs8*	regulator of G-protein signaling (RGS) protein	-	deletion	-	-	-	-	rice leaves (cultivar CO-39)	[104]
MGG_04719	Guy11	*mip11*	scaffolding protein, MoRgs7-interacting protein	fungal development and pathogenicity	deletion	+	+	+	+	rice (cv. CO39)	[106]
**The high-affinity cyclic adenosine monophosphate-dependent protein kinase A (cAMP/PKA) signaling**											
MGG_06368	70-15, 70-6, Guy11	*cpka*	catalytic subunit of the cAMP-dependent protein kinase	appressorium penetration and fungal pathogenicity	deletion	NA	NA	+	+	rice (cv. S-201 and cv. CO39) and barley (cv. Bonanza)	[108]

MGG_07335	B157	*rpka*	regulatory subunit of cAMP/PKA	fungal development and pathogenicity	deletion	+	+	+	+	rice (cv.CO39) and barley (cv. Express)	[109]
MGG_05664	B157	*pdeh*	high-affinity phosphodiesterase	fungal development and pathogenicity, regulation of cAMP levels	deletion	+	+	+	+	rice (cv. Tongil and CO39)	[110]
MGG_07707	B157	*pdel*	low-affinity cAMP phosphodiesterase	minor role	deletion	+	+	+	+	rice (cv. Tongil and CO39)	[110]
MGG_09700	Guy11	*yvh1*	protein phosphatase	fungal development and pathogenicity	deletion	+	+	+	+	rice (cv. CO39)	[111]
MGG_09898	70-15	*mac1*	adenylate cyclase, membrane-bound enzyme	fungal development and pathogenicity	deletion	+	+	+	+	rice (cv.S-201)	[112]
MGG_01722	Ku80	*cap1*	adenylate cyclase-associated protein	fungal development and pathogenicity	deletion	+	+	+	+	rice seedlings (cv. CO-39)	[113]
MGG_04708	Guy11	*som1*	pathogenicity-related gene	fungal development and pathogenicity	T-DNA insertion and deletion	+	+	+	+	barley (cv. Golden Promise) and (rice cv. CO-39)	[115]

MGG_00692	Guy11 and 70-15	*stu1*	APSES transcription factor	fungal development and pathogenicity	deletion	-	+	+	+	rice (cv. LTH)	[114, 115]
MGG_11346	Guy11	*cdtf1*	cAMP-dependent transcription factor gene	fungal development and pathogenicity	T-DNA insertion and deletion	+	+	+	+	barley (cv. Golden Promise) and rice (cv. CO-39)	[115]
MGG_00063	Guy11	*agl1*	glycogen amyloglucosidase	glycogen mobilisation and fungal pathogenicity	deletion	NA	-	-	+	rice seedlings (cultivar CO-39) and barley seedlings (Golden Promise)	[116]
MGG_01819	Guy11	*gph1*	glycogen phosphorylase	glycogen mobilisation and fungal pathogenicity	deletion	NA	-	-	+	rice seedlings (cultivar CO-39) and barley seedlings (Golden Promise)	[116]
**MAPK cascade**											
MGG_09565	Guy11	*pmk1*	pathogenicity MAP-kinase 1, the mitogen-activated protein kinase (MAPK)	appressorium formation and fungal pathogenicity	deletion	-	+	+	+	rice (cv. CO39) and barley (cv. Golden Promise)	[117]
MGG_04943	Guy11	*mps1*	mitogen-activated protein kinase (MAPK)	appressorium penetration and fungal virulence, cell wall integrity	deletion	+	+	+	+	rice (cv. CO39)	[118]

MGG_01822	TH3	*osm1*	mitogen-activated protein kinase (MAPK)	osmotic sensitivity, accumulation of the compatible solute arabitol	deletion	-	-	+	-	rice (cv. CO39)	[119]
MGG_00501	Guy11	*msn2*	transcriptional activator with the typical C2H2 zinc-finger DNA-binding domains	fungal development and pathogenicity	deletion	+	+	+	+	rice (cv. CO-39) and barley (cv. Four-arris)	[120]
MGG_14847	70-15	*mst11*	MAPKKK (MST11-MST7-PMK1 cascade)	fungal development and pathogenicity	deletion	-	+	+	+	rice (cv. Nipponbare) and barley (cv. Golden Promise)	[121]
MGG_00800	70-15	*mst7*	MAPKK (MST11-MST7-PMK1 cascade)	fungal development and pathogenicity	deletion	-	+	+	+	rice (cv. Nipponbare) and barley (cv. Golden Promise)	[122]
MGG_04236	Guy11	*trx2*	thioredoxin gene	fungal development and pathogenicity	deletion	+	+	+	+	rice seedlings (cv. CO-39)	[122]
MGG_00883	KJ201	*mck1*	MAPKKK (MCK1-MKK1-MPS1 cascade)	fungal development and pathogenicity, cell wall integrity	T-DNA insertion and deletion	+	+	+	+	rice seedlings (cv. Nagdong)	[123]
MGG_06482	Guy11	*mkk1*	MAPKK (MCK1-MKK1-MPS1 cascade)	fungal development and pathogenicity, cell wall integrity, osmotic stress response	deletion	+	+	+	+	rice (cv. Nipponbare) and barley (cv. Golden Promise)	[124]

MGG_12821	Guy11	*mst20*	p21-activated kinase (PAK) kinase	conidiation, redundant function	deletion	+	+	-	-	rice (cv. CO39) and barley (cv. Golden Promise)	[125]
MGG_06320	Guy11	*chm1*	p21-activated kinase (PAK) kinase	appressorium formation and penetration, fungal pathogenicity	deletion	+	+	+	+	rice (cv. CO39) and barley (cv. Golden Promise)	[125]
MGG_04100	Guy11	*sep1*	Mitotic Exit Network (MEN) kinase	fungal development and pathogenicity	deletion	+	+	+	+	rice seedlings (cv. CO39)	[126]
MGG_02757	Guy11	*dbf2*	MEN kinase pathway component	fungal development and pathogenicity	deletion	+	+	+	+	rice seedlings (cv. CO39)	[126]
MGG_03151	Guy11	*mob1*	MEN kinase pathway component	fungal development and pathogenicity	deletion	+	+	+	+	rice seedlings (cv. CO39)	[126]
MGG_12958	Guy11&70-15	*mst12*	Cys2-His2 (C2H2) zinc finger protein	fungal pathogenicity	deletion	-	-	-	+	barley and rice (cv.CO-39)	[128]
MGG_01285	Guy11	*tpc1*	transcription factor for polarity control 1, Zn(II)2Cys6 transcriptional regulator	fungal development and pathogenicity	deletion and T-DNA insertion	+	+	+	+	rice leaves and roots	[129]
MGG_06971	Ku80	*sfl1*	transcription factor	fungal conidiation and pathogenicity, heat tolerance	deletion	-	+	-	+	rice (cv. Nipponbare) and barley (cv. Golden Promise)	[130]

MGG_12337	Guy11	*gas1*	gEgh16 homologs expressed in appressorium stage	appressorial penetration and fungal pathogenicity	deletion	-	-	+	+	rice (cv. CO39)	[131]
MGG_04201	Guy11	*gas2*	gEgh16 homologs expressed in appressorium stage	appressorial penetration and fungal pathogenicity	deletion	-	-	+	+	rice (cv. CO39)	[131]
MGG_08600	70-15	*pic5*	PMK1-interacting clone	appressorium differentiation and fungal pathogenicity	deletion	+	+	+	+	rice (cv. Nipponbare) and barley (cv. Golden Promise)	[132]
MGG_01204	Guy11 and 70-15	*mig1*	MADS-box transcription factor	fungal development and pathogenicity	deletion	-	+	+	+	rice (cv. cv. Nipponbare) and barley (cv. Golden Promise)	[133]
MGG_09869	Guy11	*swi6*	APSES family transcription factor	fungal development and pathogenicity	deletion	+	+	+	+	rice (cv. CO-39)	[134]
MGG_12122	Ku80	*gsk1*	glycogen synthase kinase	fungal development and pathogenicity	deletion	+	+	+	+	barley seedlings (cv. CDC Silky) and rice seedlings (cv. CO39)	[135]
MGG_08850	Guy11	*gti1*	GTI1 transcription factor	cell wall integrity, conidiation, and plant infection	deletion	+	+	+	+	rice seedlings (cv. CO-39)	[136]
**Monomeric GTPase modules (Ras superfamily)**											
MGG_09499	70-15	*ras1*	GTP-binding protein	minor role in conidiation	deletion and transformant	-	+	-	-	rice (cv. CO39 and Nipponbare)	[139]

MGG_06154	70-15	*ras2*	GTP-binding protein	fungal development and pathogenicity	deletion and transformant (yeast gap repair method)	+	+	+	+	rice (cv. CO39 and Nipponbare)	[139]
MGG_03846	Guy11	*smo1*	Ras GTPase-activating protein	fungal development and pathogenicity	deletion	+	+	+	+	rice (cv.CO-39)	[140]
MGG_01287	P131	*ram1*	farnesyltransferase β-subunit	fungal development and pathogenicity, regulating localization of RAS proteins	deletion	+	+	+	+	rice seedlings (cv. LTH) and barley leaves (cv. E9)	[141]
MGG_08255	70-15	*ral2*	kelch domain-containing protein	fungal development and pathogenicity	deletion	+	+	+	+	rice (cv. CO39)	[142]
MGG_02731	70-15 and Guy11	*rac1*	small GTPase, Rho-family GTPase	fungal development and pathogenicity	deletion	+	+	+	+	rice and barley (cv. Jinchang 1316) seedlings	[143]
MGG_00466	CP987	*cdc42*	small GTPase, Rho-family GTPase	fungal development and pathogenicity	deletion	-	+	+	+	rice (cv. CO39) and barley (cv. Jinchang 1316) seedlings	[144]
MGG_02457	KJ201	*rho2*	small GTPase, Rho-family GTPase	appressorium development and pathogenicity	deletion	-	-	+	+	rice (cv. Nakdongbyeo)	[145]

MGG_10323	70-15	*rho3*	small GTPase, Rho-family GTPase	fungal development and pathogenicity	deletion	-	+	+	+	rice (cv. CO39)	[146]
MGG_04186	Δku70 and Δku80	*rga1*	Rho GTPase activating protein (Rho GAP) gene	fungal development	deletion	-	+	+	-	rice and barley (cv. Jinchang 1316) seedlings	[147]
MGG_06390	Δku70 and Δku80	*rga2*	Rho GAP gene	redundant function	deletion	-	+	-	-	rice and barley (cv. Jinchang 1316) seedlings	[147]
MGG_03048	Δku70 and Δku80	*rga3*	Rho GAP gene	-	deletion	-	-	-	-	rice and barley (cv. Jinchang 1316) seedlings	[147]
MGG_09531	Δku70 and Δku80	*rga4*	Rho GAP gene	fungal development, redundant function	deletion	+	+	+	-	rice and barley (cv. Jinchang 1316) seedlings	[147]
MGG_09303	Δku70 and Δku80	*rga5*	Rho GAP gene	redundant function	deletion	-	+	-	-	rice and barley (cv. Jinchang 1316) seedlings	[147]
MGG_09275	Δku70 and Δku80	*rga6*	Rho GAP gene	-	deletion	-	-	-	-	rice and barley (cv. Jinchang 1316) seedlings	[147]
MGG_04006	Δku70 and Δku80	*rga7*	Rho GAP gene	redundant function	deletion	-	+	-	-	rice and barley (cv. Jinchang 1316) seedlings	[147]

MGG_04377	Δku70 and Δku80	*lrg1*	Rho GTPase activating protein (Rho GAP) gene	fungal development and pathogenicity	deletion	+	+	+	+	rice and barley (cv. Jinchang 1316) seedlings	[147]
MGG_04438	Guy11	*arf6*	ADP ribosylation factor (Arf) small GTPase family protein	fungal growth and conidiation	deletion	+	+	-	-	rice (cv. CO-39)	[148]
MGG_01574	Guy11	*arl1*	ADP ribosylation factor (Arf) small GTPase family protein	vegetative growth, normal vesicle trafficking, fungal pathogenicity	deletion	+	-	+	+	rice (cv. CO-39)	[149]
MGG_08859	Guy11	*arl3*	ADP ribosylation factor (Arf) small GTPase family protein	vegetative growth	deletion	+	-	-	-	rice (cv. CO-39)	[149]
MGG_04976	Guy11	*arl8*	ADP ribosylation factor (Arf) small GTPase family protein	-	deletion	-	-	-	-	rice (cv. CO-39)	[149]
MGG_10676	Guy11	*cin4*	ADP ribosylation factor (Arf) small GTPase family protein	fungal growth, conidiation and pathogenicity, normal vesicle trafficking	deletion	+	+	+	+	rice (cv. CO-39)	[149]
MGG_06362	Guy11	*sar1*	ADP ribosylation factor (Arf) small GTPase family protein	fungal growth	ΔMoarf1/CPR mutant (nitrate reductase promoter)	+	NA	NA	-	rice (cv. CO-39)	[149]

MGG_12887	Guy11	*arf1*	ADP ribosylation factor (Arf) small GTPase family protein	fungal growth	ΔMosar1/CPR mutant (nitrate reductase promoter)	+	NA	NA	-	rice (cv. CO-39)	[149]
MGG_00852	Guy11	*gga1*	Arf-interacting Gga protein	fungal development and pathogenicity	deletion	-	+	NA	+	rice (cv. CO-39)	[149]
MGG_01472	Guy11	*glo3*	ADP ribosylation factor (Arf) GAP protein	fungal development and pathogenicity	deletion	+	+	+	+	rice (cv. CO-39)	[150]
MGG_08144	Guy11	*ypt7*	Rab GTPase	fungal development and pathogenicity	deletion	+	+	NA	+	barley (cv. Golden Promise) and rice (cv. CO39)	[151]
**Target of Rapamycin (TOR) signaling pathway (Negative-acting regulatory pathways)**											
MGG_06050	Guy11	*asd*4	GATA transcription factor	intracellular glutamine level regulation, TOR inhibition of appressorium formation	deletion	+	-	+	-	rice (cv. CO39)	[153]
MGG_03911	Guy11	*ppe1*	serine/threonine protein phosphatase	fungal development and pathogenicity, cell wall integrity	deletion	+	+	-	+	rice (cv. CO39)	[154]
MGG_12709	Guy11	*sap1*	MoPpe1 associated protein	fungal pathogenicity, cell wall integrity	deletion	+	+	+	+	rice (cv. CO39)	[154]

MGG_00478	Guy11	*tip41*	Tap42-interacting protein 41, type 2A phosphatase activator TIP41	fungal development and pathogenicity, autophagosome formation and nonspecific autophagy	deletion	+	+	-	+	rice (cv. CO39)	[155]
MGG_11241	B157	*whi2*	protein with a SKP1/BTB/POZ domain	fungal development and pathogenicity	T-DNA insertion and deletion	+	+	+	+	rice (cv. CO-39)	[156]
MGG_03646	B157	*psr1*	phosphatase	fungal development and pathogenicity	deletion	+	+	+	+	rice (cv. CO-39)	[156]
MGG_08120	Guy11	*imp1*	integral membrane protein	biotrophic interface longevity, membrane trafficking, V-ATPase assembly, organelle acidification and autophagy induction	T-DNA insertion and deletion	+	NA	+	+	rice (cv. CO39)	[157]
**Ubiquitination cascade**											
MGG_01282	70-15	NA	polyubiquitin encoding gene	fungal development and pathogenicity	deletion	+	+	+	+	barley and rice seedlings	[159]

MGG_01756	Guy11	*rad6*	ubiquitin-conjugating enzyme (E2)	fungal development and pathogenicity	deletion	+	+	+	+	rice (cv. CO39) and barley leaves	[160]
MGG_00139	Guy11	*bre1*	ubiquitin ligase (E3)	fungal development and pathogenicity	deletion	+	+	-	+	rice (cv. CO39) and barley leaves	[160]
MGG_13171	Guy11	*ubr1*	ubiquitin ligase (E3)	fungal development and pathogenicity, conidial adhesion and germination	deletion	-	+	+	+	rice (cv. CO39) and barley leaves	[160]
MGG_04175	Guy11	*rad18*	ubiquitin ligase (E3)	minor roles	deletion	-	+	-	-	rice (cv. CO39) and barley leaves	[160]
MGG_04978	B157	*skp1*	component of the SCF (Skp1-Cullin 1-F-box) E3 ubiquitin ligase complex	fungal development and pathogenicity	deletion	+	+	+	+	rice (cv. CO39) and barley leaves	[161]
MGG_13065	Guy11	*grr1*	F-box protein, specific adaptors to E3 ubiquitin ligases	fungal development and virulence	deletion	+	+	+	+	rice (cv. CO39) and barley leaves	[162]

MGG_12163	KJ201	*cue1*	the ubiquitin system component cue	fungal conidiation and pathogenicity, translocation of cytoplasmic effectors	deletion	-	+	-	+	rice (cv. Nakdongbyeo)	[163]
MGG_00768	Guy11	*fbx15*	F-box protein	fungal development and pathogenicity, circadian rhythm regulation	deletion	+	+	+	+	barley leaves (cv. Golden Promise)	[163]
MGG_04957	Guy11	*ubp4*	deubiquitinating enzyme	fungal development and pathogenicity, deubiquitination	T-DNA insertion and deletion	+	+	+	+	rice (cv. CO-39) and barley (cv. Golden Promise)	[165]
MGG_03527	Guy11	*ubp8*	deubiquitinating enzyme	fungal development and pathogenicity, carbon catabolite repression	deletion	+	+	+	+	rice (cv. CO-39)	[166]
MGG_05737	P131	*smt3*	small ubiquitin-like modifier (SUMO) pathway component	fungal development and pathogenicity, stress response	deletion	+	+	-	+	barley seedlings (Hordeum vulgare cv E9) and rice seedlings (Oryza sativa japonica cv LTH)	[167]

MGG_01669	P131	*aos1*	E1-activating enzyme gene, SUMO-activating enzyme	fungal development and pathogenicity, stress response	deletion	+	+	-	+	barley seedlings (Hordeum vulgare cv E9) and rice seedlings (Oryza sativa japonica cv LTH)	[167]
MGG_06733	P131	*uba2*	E1-activating enzyme gene, SUMO-activating enzymes	fungal development and pathogenicity, stress response	deletion	+	+	-	+	barley seedlings (Hordeum vulgare cv E9) and rice seedlings (Oryza sativa japonica cv LTH)	[167]
MGG_00970	P131	*ubc9*	E2 conjugating ligase gene, SUMO-conjugating enzyme	fungal development and pathogenicity, stress response	deletion	+	+	-	+	barley seedlings (Hordeum vulgare cv E9) and rice seedlings (Oryza sativa japonica cv LTH)	[167]
MGG_08837	P131	*siz1*	E3 ligase gene, SUMO ligases	fungal development and pathogenicity, stress response	deletion	+	+	-	+	barley seedlings (Hordeum vulgare cv E9) and rice seedlings (Oryza sativa japonica cv LTH)	[167]

(e) Multi-functional genes involved in different aspects of *M. oryzae* biology.

Gene code	Fungal Reference Strain	Mutant Name	Gene Full Name/ Encoding Protein	Main Functions	Mutant Type	Mutant Phenotypes				Tested Hosts	Reference
						Hyphal Growth	Conidia Growth/ Development	Appressoria Formation/ Development	Virulence		
**Transcription factors (Zn2Cys6 transcription factor)**											
MGG_17841	70-15	*gpf1*	Zn2Cys6 transcription factor gene, growth and pathogenicity regulatory factor 1	fungal development and pathogenicity	deletion	+	+	+	+	barley (Hordeum vulgare) and rice (Oryza sativa cv CO39)	[170]
MGG_15023	70-15	*cnf2*	Zn2Cys6 transcription factor gene, conidial production negative regulatory factor 2	fungal development and pathogenicity	deletion	NA	+	-	+	barley (Hordeum vulgare) and rice (Oryza sativa cv CO39)	[170]
MGG_07063	70-15	*gcc1*	Zn2Cys6 transcription factor gene, growth, conidiation and cell wall regulatory factor 1	fungal development and pathogenicity	deletion	+	+	-	+	barley (Hordeum vulgare) and rice (Oryza sativa cv CO39)	[170]

MGG_07149	70-15	*gta1*	Zn2Cys6 transcription factor gene, growth and tolerance to acidic stress regulatory factor 1	fungal development and pathogenicity	deletion	+	+	-	+	barley (Hordeum vulgare) and rice (Oryza sativa cv CO39)	[170]
MGG_02962	70-15	*cnf1*	Zn2Cys6 transcription factor gene, conidial production negative regulatory factor 1	fungal development and pathogenicity	deletion	NA	+	+	+	barley (Hordeum vulgare) and rice (Oryza sativa cv CO39)	[170]
MGG_17623	70-15	*pcf1*	Zn2Cys6 transcription factor gene, pathogenicity and conidiation regulatory factor 1	fungal development and pathogenicity	deletion	NA	+	+	+	barley (Hordeum vulgare) and rice (Oryza sativa cv CO39)	[170]
MGG_05659	70-15	*cca1*	Zn2Cys6 transcription factor gene, conidiation, conidial germination and appressorium formation required transcription factor 1	fungal development and pathogenicity	deletion	NA	+	+	+	barley (Hordeum vulgare) and rice (Oryza sativa cv CO39)	[170]

MGG_12349	70-15	*conx1*	Zn2Cys6 transcription factor gene, conidiation required transcription factor x1	fungal development and pathogenicity	deletion	NA	+	-	+	barley (Hordeum vulgare) and rice (Oryza sativa cv CO39)	[170]
MGG_05343	KJ201	*cod1*	conidiation-related Zn (II)2Cys6 transcription factor gene	fungal development and pathogenicity	deletion	+	+	+	+	rice seedlings (cv. Nakdongbyeo)	[169]
MGG_09263	KJ201	*cod2*	conidiation-related Zn (II)2Cys6 transcription factor gene	fungal conidiation and pathogenicity	deletion	-	+	-	+	rice seedlings (cv. Nakdongbyeo)	[169]
MGG_01553	Guy11	*leu1*	isopropylmalate isomerase	leucine biosynthesis, fungal development and pathogenicity	deletion	+	+	+	+	rice (cv. CO39) or barley (cv. Golden Promise) leaves	[171]
MGG_05223	Guy11	*leu2*	leucine biosynthesis pathway gene	leucine biosynthesis, fungal development and pathogenicity	deletion	+	+	+	+	rice (cv. CO39) or barley (cv. JZ-8)	[171]
MGG_00672	Guy11	*leu3*	a leucine-associated Zn2Cys6-type transcription factor	leucine biosynthesis, fungal development and pathogenicity	deletion	-	-	+	+	rice (cv. CO39) or barley (cv. JZ-8)	[171]

MGG_13485	Guy11	*leu4*	leucine biosynthesis pathway gene	leucine biosynthesis, fungal development and pathogenicity	deletion	-	+	+	+	rice (cv. CO39) or barley (cv. JZ-8)	[171]
**Transcription factors (Cys2-His2 (C2H2) zinc finger protein family)**											
MGG_05287	70-15	*con7*	Cys2-His2 (C2H2) zinc finger protein	fungal development and pathogenicity	deletion	+	+	NA	+	barley and rice (cv.CO-39)	[172, 175]
MGG_02775	70-15	*conx2*	Cys2-His2 (C2H2) zinc finger protein	fungal development and pathogenicity	deletion	-	+	NA	+	barley and rice (cv.CO-39)	[175]
MGG_01017	70-15	*conx3*	Cys2-His2 (C2H2) zinc finger protein	fungal development and pathogenicity	deletion	-	+	+	+	barley and rice (cv.CO-39)	[175]
MGG_02055	70-15	*conx4*	Cys2-His2 (C2H2) zinc finger protein	conidiation	deletion	-	+	-	-	barley and rice (cv.CO-39)	[175]
MGG_07339	70-15	*conx5*	Cys2-His2 (C2H2) zinc finger protein	fungal development and pathogenicity	deletion	-	+	-	+	barley and rice (cv.CO-39)	[175]
MGG_03133	70-15	*conx6*	Cys2-His2 (C2H2) zinc finger protein family	conidiation	deletion	-	+	-	-	barley and rice (cv.CO-39)	[175]
MGG_01127	70-15	*conx7*	Cys2-His2 (C2H2) zinc finger protein	conidiation	deletion	-	+	-	-	barley and rice (cv.CO-39)	[175]

MGG_06848	70-15	*conx8*	Cys2-His2 (C2H2) zinc finger protein	conidiation	deletion	-	+	-	-	barley and rice (cv.CO-39)	[175]
MGG_06575	70-15	*conx9*	Cys2-His2 (C2H2) zinc finger protein	conidiation	deletion	-	+	-	-	barley and rice (cv.CO-39)	[175]
MGG_08493	70-15	*conx10*	Cys2-His2 (C2H2) zinc finger protein	conidiation	deletion	-	+	-	-	barley and rice (cv.CO-39)	[175]
MGG_03030	70-15	*conx11*	Cys2-His2 (C2H2) zinc finger protein	conidiation	deletion	-	+	-	-	barley and rice (cv.CO-39)	[175]
MGG_03977	70-15	*cos1*	Cys2-His2 (C2H2) zinc finger protein	conidiation	deletion	-	+	NA	-	barley and rice (cv.CO-39)	[175]
MGG_10595	70-15	*gcf2*	Cys2-His2 (C2H2) zinc finger protein	fungal development and pathogenicity	deletion	+	+	-	+	barley and rice (cv.CO-39)	[175]
MGG_11252	70-15	*gcf3*	Cys2-His2 (C2H2) zinc finger protein	fungal development and pathogenicity	deletion	+	+	-	+	barley and rice (cv.CO-39)	[175]
MGG_06328	70-15	*gcf4*	Cys2-His2 (C2H2) zinc finger protein	fungal development	deletion	+	+	-	-	barley and rice (cv.CO-39)	[175]
MGG_17953	70-15	*gcf5*	Cys2-His2 (C2H2) zinc finger protein	fungal development	deletion	+	+	-	-	barley and rice (cv.CO-39)	[175]

MGG_03451	70-15	*gcf6*	Cys2-His2 (C2H2) zinc finger protein	fungal development and pathogenicity	deletion	+	+	-	+	barley and rice (cv.CO-39)	[175]
MGG_06364	70-15	*gcf7*	Cys2-His2 (C2H2) zinc finger protein	fungal development	deletion	+	+	-	-	barley and rice (cv.CO-39)	[175]
MGG_15393	70-15	*gcf8*	Cys2-His2 (C2H2) zinc finger protein	fungal development	deletion	+	+	-	-	barley and rice (cv.CO-39)	[175]
MGG_00373	70-15	*gcp1*	Cys2-His2 (C2H2) zinc finger protein	fungal development and pathogenicity	deletion	+	+	-	+	barley and rice (cv.CO-39)	[175]
MGG_02474	70-15	*gpf2*	Cys2-His2 (C2H2) zinc finger protein	fungal development and pathogenicity	deletion	+	-	-	+	barley and rice (cv.CO-39)	[175]
MGG_07011	70-15	*gpf3*	Cys2-His2 (C2H2) zinc finger protein	fungal development and pathogenicity	deletion	+	-	-	+	barley and rice (cv.CO-39)	[175]
MGG_05133	70-15	*crz1*	Cys2-His2 (C2H2) zinc finger protein	fungal development and pathogenicity, calcineurin-responsive transcription factor	deletion	+	+	-	+	barley and rice (cv.CO-39)	[173]
MGG_04699	70-15	*flbc*	Cys2-His2 (C2H2) zinc finger protein	fungal development and pathogenicity, calcineurin-responsive transcription factor	deletion	+	+	+	+	barley and rice (cv.CO-39)	[175]

MGG_00504	70-15	*nsdc*	Cys2-His2 (C2H2) zinc finger protein	fungal development	deletion	+	+	-	-	barley and rice (cv.CO-39)	[175]
MGG_02505	70-15	*rei1*	Cys2-His2 (C2H2) zinc finger protein	fungal development and pathogenicity	deletion	+	+	-	+	barley and rice (cv.CO-39)	[175]
MGG_04456	70-15	*zap1*	Cys2-His2 (C2H2) zinc finger protein	conidiation	deletion	-	+	-	-	barley and rice (cv.CO-39)	[175]
MGG_12958	70-15	*mst12*	Cys2-His2 (C2H2) zinc finger protein	fungal pathogenicity	deletion	-	-	-	+	barley and rice (cv.CO-39)	[175]
MGG_09200	70-15	*tdg1*	Cys2-His2 (C2H2) zinc finger protein	fungal development and pathogenicity	deletion	-	+	-	+	barley and rice (cv.CO-39)	[175]
MGG_14931	70-15	*vrf1*	Cys2-His2 (C2H2) zinc finger protein	fungal development and pathogenicity	deletion	-	-	+	+	barley and rice (cv.CO-39)	[175]
MGG_01776	70-15	*vrf2*	Cys2-His2 (C2H2) zinc finger protein	fungal development and pathogenicity	deletion	-	-	+	+	barley and rice (cv.CO-39)	[175]
MGG_00660	70-15	*zfp1*	Cys2-His2 (C2H2) zinc finger protein	fungal pathogenicity	deletion	-	-	-	+	barley and rice (cv.CO-39)	[175]
MGG_04865	70-15	*zfp2*	Cys2-His2 (C2H2) zinc finger protein	fungal development	deletion	+	-	-	-	barley and rice (cv.CO-39)	[175]

MGG_05714	70-15	*zfp3*	Cys2-His2 (C2H2) zinc finger protein	fungal development	deletion	+	-	-	-	barley and rice (cv.CO-39)	[175]
MGG_09780	70-15	*zfp4*	Cys2-His2 (C2H2) zinc finger protein	no development or virulence functions	deletion	-	-	-	-	barley and rice (cv.CO-39)	[175]
MGG_14806	70-15	*zfp5*	Cys2-His2 (C2H2) zinc finger protein	fungal development and pathogenicity	deletion	+	-	-	+	barley and rice (cv.CO-39)	[175]
MGG_15508	70-15	*zfp6*	Cys2-His2 (C2H2) zinc finger protein	fungal pathogenicity	deletion	-	-	-	+	barley and rice (cv.CO-39)	[175]
MGG_00080	70-15	*zfp7*	Cys2-His2 (C2H2) zinc finger protein	no development or virulence functions	deletion	-	-	-	-	barley and rice (cv.CO-39)	[175]
MGG_02036	70-15	*zfp8*	Cys2-His2 (C2H2) zinc finger protein	fungal pathogenicity	deletion	-	-	-	+	barley and rice (cv.CO-39)	[175]
MGG_03581	70-15	*zfp9*	Cys2-His2 (C2H2) zinc finger protein	fungal development	deletion	+	-	-	-	barley and rice (cv.CO-39)	[175]
MGG_04328	70-15	*zfp10*	Cys2-His2 (C2H2) zinc finger protein	fungal development and pathogenicity	deletion	+	-	-	+	barley and rice (cv.CO-39)	[175]
MGG_05501	70-15	*zfp11*	Cys2-His2 (C2H2) zinc finger protein	fungal pathogenicity	deletion	-	-	-	+	barley and rice (cv.CO-39)	[175]

MGG_07013	70-15	*zfp12*	Cys2-His2 (C2H2) zinc finger protein	no development or virulence functions	deletion	-	-	-	-	barley and rice (cv.CO-39)	[175]
MGG_07269	70-15	*zfp13*	Cys2-His2 (C2H2) zinc finger protein	no development or virulence functions	deletion	-	-	-	-	barley and rice (cv.CO-39)	[175]
MGG_11925	70-15	*zfp14*	Cys2-His2 (C2H2) zinc finger protein	fungal development	deletion	-	+	+	-	barley and rice (cv.CO-39)	[175]
MGG_15991	70-15	*zfp15*	Cys2-His2 (C2H2) zinc finger protein	fungal development	deletion	-	+	-	-	barley and rice (cv.CO-39)	[175]
MGG_11201	Ku80	*crea*	carbon catabolite repressor, Cys2-His2 (C2H2) zinc finger protein	fungal development and pathogenicity	deletion	+	+	+	+	rice (cv. CO-39)	[174]
**Transcription factors (the basic leucine zipper (bZIP) transcription factor)**											
MGG_08212	Guy11	*atf1*	basic leucine zipper (bZIP) transcription factor	fungal oxidative stress responses and virulence	deletion	+	-	-	+	rice leaves (cultivar CO-39)	[176]
MGG_12814	Guy11	*ap1*	bZIP transcription factor	fungal development and pathogenicity	deletion	+	+	+	+	rice leaves (cultivar CO-39)	[177]
MGG_14561	Guy11	*metr/bizp22*	bZIP transcription factor	fungal development and pathogenicity	deletion	+	+	-	+	rice leaves	[179]

MGG_09010	Guy11	*hac1*	bZIP transcription factor	fungal development and pathogenicity	deletion	+	+	-	+	rice leaves	[179]
MGG_06131	Guy11	*bzip10*	bZIP transcription factor	fungal development and pathogenicity	deletion	+	+	+	+	rice leaves	[179]
MGG_01569	Guy11	*ycp4*	flavodoxin-like protein	fungal development and pathogenicity	deletion	+	+	+	+	rice leaves (cultivar CO-39)	[180]
MGG_02006	KJ201	*bzip4*	bZIP transcription factor	fungal pathogenicity	deletion	-	-	-	+	rice seedlings (cv. Nakdongb yeo)	[178]
MGG_03288	KJ201	*bzip7*	bZIP transcription factor	fungal development and pathogenicity	deletion	+	+	-	+	rice seedlings (cv. Nakdongb yeo)	[178]
MGG_05016	KJ201	*bzip11*	bZIP transcription factor	fungal development and pathogenicity	deletion	+	-	+	+	rice seedlings (cv. Nakdongb yeo)	[178]
MGG_05959	KJ201	*bzip13*	bZIP transcription factor	fungal development and pathogenicity	deletion	+	+	+	+	rice seedlings (cv. Nakdongb yeo)	[178]

Other transcription factors

MGG_04853	KJ201	*hox1*	homeobox transcription factor	hyphal growth	deletio n	+	NA	NA	NA	rice seedlings (cv. Nakdongb yeo)	[182]

MGG_00184	KJ201	*hox2*	homeobox transcription factor	conidiation	deletion	NA	+	-	-	rice seedlings (cv. Nakdongbyeo)	[182]
MGG_06285	KJ201	*hox4*	homeobox transcription factor	conidiation	deletion	NA	+	NA	NA	rice seedlings (cv. Nakdongbyeo)	[182]
MGG_11712	KJ201	*hox6*	homeobox transcription factor	hyphal growth	deletion	+	-	-	-	rice seedlings (cv. Nakdongbyeo)	[182]
MGG_12865	KJ201	*hox7*	homeobox transcription factor	appressoria formation	deletion	-	NA	+	+	rice seedlings (cv. Nakdongbyeo)	[182]
MGG_06258	KJ201	*fkh1*	forkhead-box (FOX) transcription factor	fungal development and pathogenicity	deletion	+	+	+	+	rice seedlings (cv. Nakdongbyeo)	[183]
MGG_06422	KJ201	*hcm1*	FOX transcription factor	mycelial growth and conidial germination	deletion	+	+	-	-	rice seedlings (cv. Nakdongbyeo)	[183]
MGG_05709	70-15	*crf1*	basic helix–loop–helix (bHLH) transcription factor	fungal development and pathogenicity	deletion	+	+	+	+	barley (cv. ZJ-8) and rice (cv. CO39)	[184, 185]
MGG_10150	Guy11	*pacc*	transcription factor	fungal development and pathogenicity	deletion	+	+	NA	+	rice and barley seedlings	[187]

MGG_10197	70-15	*tra1*	transcription factor	fungal development and pathogenicity	deletion	+	+	+	+	rice seedling (cv. CO-39) and barley seedling (cv. Golden Promise)	[188]
MGG_00501	70-15	*tdg2*	transcription factor	fungal adhesion and virulence	deletion	NA	+	+	+	rice seedling (cv. CO-39) and barley seedling (cv. Golden Promise)	[188]
MGG_03148	70-15	*tdg4*	Tra1-dependent gene	fungal sporulation and virulence	deletion	NA	+	+	+	rice seedling (cv. CO-39) and barley seedling (cv. Golden Promise)	[188]
**Kinases**											
MGG_00446	Ku80	*ckb1*	serine/threonine kinase CKb regulatory subunit	fungal development and pathogenicity	deletion	+	+	+	+	rice (cv. CO-39)	[190]
MGG_05651	Ku80	*ckb2*	serine/threonine kinase CKb regulatory subunit	fungal development and pathogenicity	deletion	+	+	+	+	rice (cv. CO-39)	[190]

MGG_11326	Guy11	*ark1*	actin-regulating kinase, the serine/ threonine protein kinase (SPK)	endocytosis and actin cytoskeleton organization, fungal development and pathogenicity	deletion	+	+	+	+	barley leaves (cv. Four-arris) and rice seedlings (cv. CO39)	[191]
MGG_06358	Guy11	*abp1*	MoArk1 kinase-interacting actin binding protein	fungal development and pathogenicity, endocytosis, actin cytoskeleton dynamics regulation	deletion	+	NA	+	+	rice seedlings (cv. CO-39)	[192]
MGG_06399	KJ201	*yak1*	dual-specificity tyrosine-regulated protein kinase	fungal development and virulence	deletion	+	+	+	+	rice (cv. Nakdongbyeo)	[193]
MGG_00682	Guy11	*cks1*	cyclin dependent kinase subunit	fungal development and pathogenicity, chitin and glucan synthase activity	deletion	+	+	+	+	rice (cv. CO-39)	[194]
MGG_06394	Guy11	*guk2*	atypical guanylate kinase	fungal development and pathogenicity	deletion	+	+	+	+	rice and barley	[195]
MGG_00803	70-15	*snf1*	protein kinase	sporulation and pathogenicity	deletion	+	+	+	+	rice plant	[197]
MGG_06930	Guy11	*sip2*	β-subunit in SNF1 pathway	fungal development and pathogenicity	deletion	+	+	+	+	rice seedlings (cv. CO39)	[198]

MGG_04005	Guy11	*snf4*	γ-subunit in SNF1 pathway	fungal development and pathogenicity	deletion	+	+	+	+	rice seedlings (cv. CO39)	[198]
MGG_07003	Guy11	*sak1*	Snf1-activating kinase	fungal development and pathogenicity	deletion	+	+	+	+	rice seedlings (cv. CO39)	[198]
MGG_07312	Guy11	*sln1*	histidine kinase	fungal development and pathogenicity, cell wall integrity	deletion	+	+	+	+	rice seedlings (cv. CO-39)	[202]
MGG_02665	KJ201	*pas1*	PAS-containing histidine kinase	fungal development and pathogenicity	deletion	NA	+	+	+	rice seedlings (cv. Nakdongbyeo)	[203]
MGG_01342	70-15	*hik2*	histidine kinases	CoCl2 sensitive, ion stress (CuSO4), fungal development and pathogenicity	deletion	-	+	-	+	rice (cv. CO-39)	[199]
MGG_12530	70-15	*hik3*	histidine kinases	fungal development and pathogenicity	deletion	-	+	-	+	rice (cv. CO-39)	[199]
MGG_13891	70-15	*hik4*	histidine kinases	CoCl2 sensitive, fungal development and pathogenicity	deletion	-	+	-	+	rice (cv. CO-39)	[199]

MGG_11882	70-15	*hik5*	histidine kinases	fungal development and pathogenicity, cell wall integrity	deletion	-	+	+	+	rice (cv. CO-39)	[199]
MGG_06696	70-15	*hik6*	histidine kinases	fungal development and pathogenicity	deletion	-	+	-	+	rice (cv. CO-39)	[199]
MGG_01227	70-15	*hik8*	histidine kinases	CoCl2 sensitive, fungal development and pathogenicity	deletion	-	+	+	+	rice (cv. CO-39)	[199]

Phosphatases

MGG_01690	P131	*ppg1*	serine/threonine-protein phosphatase 2A	asexual development and fungal pathogenicity	T-DNA insertion and deletion	+	+	+	+	rice seedlings (cultivar Lijiangxint uanheigu (LTH))	[204]
MGG_04637	Guy11	*cdc14*	dual-specificity phosphatases	fungal development and pathogenicity	deletion	+	+	+	+	rice (cv. CO-39)	[205]
MGG_03860	Guy11	*tps1*	trehalose-6-phosphate (T6P) synthase	carbon and nitrogen metabolism, fungal development and pathogenicity	deletion	+	+	+	+	rice seedlings (cv. CO39)	[207]

MGG_03441	70-15	*tps2*	trehalose 6-phospahte phosphatase	fungal development and pathogenicity	deletion	+	+	+	+	barley leaves	[206]
MGG_00543	Guy11	*tps3*	T6P regulatory subunit	fungal pathogenicity	deletion	-	NA	NA	+	rice seedlings (cv. CO39)	[206]
MGG_09994	KJ201	*lpp3*	lipid phosphate phosphatase	fungal development and pathogenicity	deletion	+	NA	+	+	rice seedlings (cv. Nakdongbyeo)	[208]
MGG_12462	KJ201	*lpp5*	lipid phosphate phosphatase	fungal development and pathogenicity	deletion	-	NA	+	+	rice seedlings (cv. Nakdongbyeo)	[208]
MGG_01311	P131	*pah1*	phosphatidate phosphatase	fungal development and pathogenesis, lipid metabolism	deletion the polyethylene glycol (PEG)-mediated approach, hygromycin B	+	+	NA	+	rice leaves (cv. Lijiangxintuanheigu)	[209]

Peroxisomal and mitochondrial related genes

MGG_09299	Guy11	*pex1*	peroxin 1 gene	fungal development and pathogenicity	T-DNA insertion and deletion	+	+	+	+	rice (cv. CO-39) and barley (cv. Golden Promise)	[217]

MGG_10840	Guy11	*pex5*	peroxisomal matrix protein, peroxisomal targeting signal 1 (PTS1) gene	fungal development and pathogenicity	deletion	+	+	+	+	rice (cv. CO39) and barley (cv. ZJ-8)	[216]
MGG_00529	Guy11	*pex6*	peroxisomal acetyl-CoA	fungal development and pathogenicity	deletion	+	+	+	+	rice (cv. CO-39)	[210]
MGG_01481	KJ201	*pex7*	PTS2 (Peroxisomal Targeting Signal 2) receptor gene	fungal development and pathogenicity	T-DNA insertion and deletion	+	+	+	+	rice leaves	[211]
MGG_08896	Guy11	*pex11A*	Pex11 Family Member	peroxisomal proliferation, fungal development and virulence	deletion	+	+	+	+	rice (cv. CO-39) and barley (cv. ZJ-8)	[212]
MGG_00157	Guy11	*pex13*	key component of the peroxisomal docking complex	fungal development and pathogenicity, peroxisome formation, lipid degradation and migration	deletion	+	+	+	+	rice (cv. CO-39) and barley (cv. ZJ-8)	[213]
MGG_01028	Guy11	*pex14*	key component of the peroxisomal docking complex	fungal development and pathogenicity, peroxisome formation, lipid degradation and migration	deletion	+	+	+	+	rice (cv. CO-39) and barley (cv. ZJ-8)	[213]

MGG_01081	Guy11	*pex14/17*	filamentous fungus-specific peroxin	fungal development and pathogenicity, fatty acid utilization, cell wall integrity	deletion	-	+	+	+	barley (cv. ZJ-8) and rice (cv. CO-39)	[214]
MGG_00971	Guy11	*pex19*	peroxisomes-related proteins	peroxisomal structure maintenance, fungal development and pathogenicity	deletion	+	+	+	+	rice seedlings (cv. CO-39) and barley (cv. ZJ-8)	[215]
MGG_01711	P131 and S1528	*pef1*	peroxisomal fission gene 1	fungal development and pathogenicity, peroxisomal fission	T-DNA insertion and deletion	+	-	+	+	rice seedling (cv. LTH) and barley seedling (cv. E9)	[218]
MGG_01721	Guy11	*pth2*	peroxisome-associated carnitine acetyltransferase	fungal development and pathogenicity	deletion	+	-	+	+	rice (cv. CO-39) and barley (cv. Golden Promise)	[220]
MGG_06199	Guy11	*pcs60*	peroxisomal-CoA synthetase	fatty acid metabolism and fungal pathogenicity	deletion	+	+	-	+	rice seedlings cv. (CO39)	[221]
MGG_02525	P131	*agt1*	peroxisomal alanine: glyoxylate aminotransferase 1	appressoria formation and pathogenicity	deletion	-	-	+	+	rice seedlings (cultivar Ribenqing) and barley (cultivar CDC Silky)	[222]

MGG_08690	Guy11	*scad2*	short-chain acyl-CoA dehydrogenase	fungal development and pathogenicity	deletion	-	+	+	+	rice (cv. CO-39)	[224]
MGG_01719	Guy11	*etfA*	electron-transferring flavoprotein (ETF) α subunit	fatty acid metabolism, fungal development and virulence	deletion	+	+	+	+	rice (cv. CO-39) and barley (cv.Golden Promise)	[226]
MGG_01744	Guy11	*etfB*	electron-transferring flavoprotein (ETF) β subunit	fatty acid metabolism, fungal development and virulence	T-DNA insertion and deletion	+	+	+	+	rice (cv. CO-39) and barley (cv.Golden Promise)	[226]
MGG_08880	Guy11	*etfdh*	electron-transferring flavoprotein (ETF) dehydrogenase	fatty acid metabolism, fungal development and virulence	deletion	+	+	+	+	rice (cv. CO-39) and barley (cv.Golden Promise)	[226]
MGG_12868	B157	*ech1*	Enoyl-CoA hydratase	conidial germination and invasive growth	deletion	+	+	+	+	barley and rice leaves	[227]
MGG_03335	Guy11	*auh1*	3-methylglutaconyl-CoA hydratase-encoding gene	fungal development and pathogenicity, mitochondrial fission/fusion balance	deletion	+	+	-	+	rice (cv. CO-39)	[228]
MGG_06075	Guy11	*fis1*	mitochondrial fission protein	fungal development and virulence	deletion	+	+	-	+	rice (cv. CO-39) and barley	[229]

MGG_06361	Guy11	*dnm1*	dynamin	fungal development and pathogenicity, peroxisomal and mitochondrial fission	deletion	+	+	+	+	rice (cv. CO-39)	[230]
MGG_01711	Guy11	*mdv1*	WD-40 repeat protein	fungal development and pathogenicity, peroxisomal and mitochondrial fission	deletion	+	+	+	+	rice (cv. CO-39)	[230]
MGG_02540	Guy11	*ivd*	isovaleryl-CoA dehydrogenase	leucine catabolism, fungal conidiation and pathogenicity	deletion	+	+	-	+	rice (cv. CO-39)	[231]
MGG_01755	Ku80	*acat1*	acetoacetyl-CoA acetyltransferase gene	fungal pathogenicity	deletion	-	-	-	+	rice (cv. CO-39) and barley (cv. Jinchang 1316)	[232]
MGG_13499	Ku80	*acat2*	acetoacetyl-CoA acetyltransferase gene	fungal development and virulence	deletion	+	-	-	+	rice (cv. CO-39) and barley (cv. Jinchang 1316)	[232]

**Other important genes in *M. oryzae* biology**											
MGG_07219	70-15	*alb1*	polyketide synthase, melanin synthesis gene	fungal melanin and pathogenicity	deletion	+	+	+	+	barley or rice leaves	[235]
MGG_05059	Guy11 and 70-15	*rsy1*	scytalone dehydratase, melanin synthesis gene	fungal melanin and pathogenicity	deletion	+	+	+	+	barley or rice leaves	[235]
MGG_02252	Guy11 and 70-15	*buf1*	1,3,8-trihydroxynaphthalene reductase, melanin synthesis gene	fungal melanin and pathogenicity	deletion	+	+	-	+	barley or rice leaves	[235]
MGG_14221	B157	*tam1*	tryptophan aminotransferase	fungal auxin/IAA biosynthesis, fungal development and pathogenicity	deletion	+	+	NA	+	rice seedlings (cv. CO39)	[237]
MGG_01892	B157	*ipd1*	indole-3-pyruvate decarboxylase	fungal auxin/IAA biosynthesis, fungal development and pathogenicity	deletion	+	+	NA	+	rice seedlings (cv. CO39)	[237]
MGG_13453	70-15	*rdrp2*	RNA interference (RNAi) gene	generation of small noncoding RNA (sRNA), fungal development and pathogenicity	deletion	+	+	NA	+	barley leaves	[239]

MGG_01294	70-15	*ago3*	RNA interference (RNAi) gene	generation of small noncoding RNA (sRNA), fungal development and pathogenicity	deletion	+	+	NA	+	barley leaves	[239]
MGG_04621	70-15	*yth1*	N6-methyladenosine (m6A)-binding protein	RNA methylation and fungal pathogenicity	deletion	-	-	-	+	rice seedlings (cv. CO39)	[242]
MGG_00637	70-15	*yth2*	N6-methyladenosine (m6A)-binding protein	RNA methylation, fungal conidiation and pathogenicity	deletion	-	+	-	+	rice seedlings (cv. CO39)	[242]
MGG_01492	70-15	*ime4*	N6-adenosine-methyltransferase	RNA methylation and fungal pathogenicity	deletion	-	-	-	+	rice seedlings (cv. CO39)	[242]
MGG_01363	70-15	*alkb1*	mRNA:N6-methyladenosine demethylase	RNA methylation and fungal pathogenicity	deletion	-	-	-	+	rice seedlings (cv. CO39)	[242]
MGG_15053	Br48	*set1*	histone H3K4 methyltransferase	fungal development and pathogenicity	deletion	+	+	+	+	wheat (cv. Norin 4, Chinese spring and Thatcher) and barley (cv. Russian No.74 and Nigrate)	[244]
MGG_05969	KJ201	*rtt109*	regulator of Ty1 transposition 109, histone acetyltransferase	fungal development and pathogenicity	deletion	+	+	-	+	rice seedlings (cv. Nagdong)	[245]

MGG_04615	KJ201	*sas3*	MYST family histone acetyltransferase	fungal development and pathogenicity	deletion	+	+	+	+	rice seedlings (cv. Nakdongbyeo)	[246]
MGG_05857	B157	*rpd3*	histone deacetylase	fungal development and pathogenicity	deletion	+	+	-	+	barley seedlings (cv. Golden Promise) and rice (cv. LTH)	[247]
MGG_04588	B157	*hst4*	histone deacetylase	fungal development and pathogenicity	deletion	+	+	-	+	barley seedlings (cv. Golden Promise) and rice (cv. LTH)	[247]
MGG_03198	Guy11 and 70-15	*tig1*	component of histone deacetylase (HDAC) transcriptional corepressor complex	fungal development and pathogenicity	deletion	+	+	-	+	rice leaves (cultivar Nipponbare or CO-39) and barley leaves	[248]
MGG_01633	Guy11 and 70-15	*hos2*	component of the TIG1 histone deacetylase complex	fungal development and pathogenicity	deletion	+	+	-	+	rice leaves (cv. Nipponbare or CO-39) and barley leaves	[248]
MGG_14558	Guy11 and 70-15	*snt1*	component of the TIG1 histoned deacetylase complex	fungal development and pathogenicity	deletion	+	+	-	+	rice leaves (cv. Nipponbare or CO-39) and barley leaves	[248]

MGG_01558	Guy11 and 70-15	*set3*	Tig1-interacting protein	fungal development and pathogenicity	deletion	NA	+	NA	+	rice leaves (cv. Nipponbare or CO-39) and barley leaves	[248]
MGG_02488	Guy11 and 70-15	*hst1*	component of the TIG1 histone deacetylase complex	fungal development and pathogenicity	deletion	+	+	-	+	rice leaves (cv. Nipponbare or CO-39) and barley leaves	[248]
MGG_04878	KJ201	*jmj1*	histone demethylase containing JmjC domain	fungal development and pathogenicity	deletion	+	+	+	+	rice seedlings (cv. Nakdongbyeo)	[249]
MGG_00750	Guy11	*nox1*	NADPH oxidase-encoding gene	production of superoxide, fungal development and virulence	deletion	+	-	+	+	rice (cv. CO-39)	[250]
MGG_06559	Guy11	*nox2*	NADPH oxidase-encoding gene	production of superoxide, fungal development and virulence	deletion	+	-	+	+	rice (cv. CO-39)	[250]
MGG_07190	Guy11	*pmt2*	O-mannosyltransferases	fungal development and virulence	deletion	+	+	+	+	rice (cv. CO-39) and barley	[251]
MGG_04427	Guy11	*pmt4*	O-mannosyltransferases	fungal development and virulence, lipid and glycogen metabolism	deletion	+	+	+	+	rice seedlings (cv. CO-39) and barleys (cv. Golden Promise)	[252]

MGG_02444	70-15	*plc1*	phospholipase C gene	fungal development and pathogenicity	deletion	+	+	+	+	rice (cv. Nagdong)	[253, 254]
MGG_05332	KJ201	*plc2*	phospholipase C gene, Ca2+-dependent membrane binding	fungal development and pathogenicity	deletion	-	+	+	+	rice (cv. Nagdong)	[253, 254]
MGG_08315	KJ201	*plc3*	phospholipase C gene, Ca2+-dependent membrane binding	fungal development and pathogenicity	deletion	-	+	+	+	rice (cv. Nagdong)	[253, 254]

(f) Genes not discussed in detail. Due to space constraints, these genes are not examined in detail here. They are ordered according to their gene codes.

Gene code	Fungal Reference Strain	Mutant Name	Gene Full Name/ Encoding Protein	Main Functions	Mutant Type	Mutant Phenotypes				Tested Hosts	Reference
						Hyphal Growth	Conidia Growth/ Development	Appressoria Formation/ Development	Virulence		
MGG_00255	KJ201	*ypel2*	yippee-like (YPEL) gene family	fungal development and pathogenicity	deletion	-	+	+	+	rice (cv. Nakdongbyo e)	[255]
MGG_00748	Guy11	*myo1*	class I myosin protein	fungal development and pathogenicity	RNAi	+	+	+	+	rice seedlings (cv. CO-39) and barley leaves (cv. Golden Promise)	[256]
MGG_00753	KJ201	*rbp9*	Ran-binding protein microtubule-organizing center	fungal development and pathogenicity, polycyclic dissemination	deletion	+	+	+	+	rice seedlings (cv. Nakdongbye o)	[257]
MGG_00874	B157	*dam1*	outer kinetochore DASH complex gene	fungal development and pathogenicity, proper segregation of chromosomes	deletion	+	+	+	+	rice (cv. CO-39)	[258]
MGG_01043	Guy11	*crec*	WD40-repeat protein	carbon repression, fungal development and pathogenicity	deletion	+	+	+	+	barley (cv. Gold Promise) and rice (cv. CO39) leaves	[259]
MGG_01057	Guy11	*ldb1*	LIM binding domain-containing protein	fungal development and pathogenicity	T-DNA insertion and deletion	+	+	+	+	rice and barley leaves	[260]

MGG_01092	Guy11	*lys20*	a homocitrate synthase	fungal development and pathogenicity	deletion	+	+	NA	+	rice seedlings (cv. CO-39)	[261]
MGG_01104	Guy11	*ilv6*	acetolactate synthase (ALS) catalytic subunit	fungal development and pathogenicity	deletion	+	+	+	+	rice and barley leaves	[262]
MGG_01185	Guy11	*rab5b*	GTP-binding protein	fungal development and pathogenicity	deletion	+	+	+	+	rice (cv. CO39) and barley (cv. Golden Promise)	[263]
MGG_01202	Ku70	*dld1*	D-lactate dehydrogenase	fungal development and pathogenicity	deletion	+	+	+	+	barley (cv. CDC silky)	[264]
MGG_01215	P131	*com1*	conidium morphology mutant 1, putative transcription regulator	fungal conidium morphology and virulence	deletion	+	+	+	+	rice and barley seedlings	[265]
MGG_01284	Guy11	*trr1*	thioredoxin reductase	fungal development and pathogenicity	deletion	-	+	+	+	rice seedlings (cv. CO-39)	[266]
MGG_01434	Ku80	*vps17*	sorting nexin	fungal development and pathogenicity	deletion	+	+	+	+	barley (cv. Four-arris) and rice (cv. CO-39)	[267]
MGG_01606	Guy11	*msdh*	methylmalonate-semialdehyde dehydrogenase	fungal development and pathogenicity	deletion	-	+	+	+	rice (cv. CO-39)	[268]

MGG_01728	Guy11	*met13*	methylenetetrahydrofolate reductases (MTHFRs)	fungal development and pathogenicity	T-DNA insertion and deletion	+	+	+	+	rice seedling (cv. CO-39) and barley seedling (cv. Golden Promise)	[269]
MGG_01743	Guy11	*cpa1*	CPSase_sm_chain and GATase domains containing protein	fungal development and pathogenicity, arginine biosynthesis	deletion	+	+	+	+	barley	[270]
MGG_01802	P131 and S1528	*chs1*	chitin synthase gene	fungal development and pathogenicity	deletion	+	+	+	+	rice leaf sheaths and barley leaves	[271]
MGG_02549	Guy11	*erl1*	Era (Escherichia coli Ras)-like GTPase	fungal development and pathogenicity	deletion	-	-	+	+	rice roots	[272]
MGG_02611	Guy11	*lys2*	l-aminoadipate-semialdehyde dehydrogenase	lysine biosynthesis, fungal development and pathogenicity	deletion	-	+	-	+	rice seedlings (cv.CO-39)	[273]
MGG_02616	70-15	*mcl1*	2-methylisocitrate lyase	fungal development and pathogenicity, methylcitrate cycle	deletion	+	+	-	+	barley (cv. ZJ-8) and rice (cv. CO-39)	[274]
MGG_02689	Guy11	*alo1*	D-Arabinono-1,4-lactone Oxidase	fungal development and pathogenicity	deletion	+	+	+	+	rice (cv. CO-39) and barley (cv. ZJ-8)	[275]

MGG_02875	Guy11	*tea1*	kelch domain-containing protein	fungal development and pathogenesis	deletion	+	+	+	+	rice seedlings (cv. CO39)	[276]
MGG_02884	Guy11	*flp1*	fungal fasciclin-like protein	fungal conidiation and pathogenicity	deletion	-	+	-	+	rice seedlings (cv. CO-39) and barley leaves (cv. ZJ-8)	[276]
MGG_02916	B157	*twl*	circadian-regulated twilight	fungal development and virulence	deletion	-	+	NA	+	barley leaf explants and rice leaf explants	[278]
MGG_02961	KJ201	*and1*	cell cortex protein	fungal asexual and sexual development and pathogenicity	deletion	-	+	+	+	rice seedlings (cv. Nakdongbyeo)	[279]
MGG_03060	Guy11	*myo2*	class II myosin	fungal development and pathogenicity, cell wall integrity	T-DNA insertion and deletion	+	+	+	+	rice seedlings (cv. CO-39) and barley leaves (cv. Golden Promise)	[280]
MGG_03065	Guy11	*vma11*	putative vacuolar ATPase c' subunit	vacuolar acidification and fungal pathogenicity	deletion	+	+	+	+	rice (cv. CO39) and barley leaves	[281]
MGG_03090	Guy11	*lag1*	sphingosine N-acyltransferase	fungal development and pathogenicity	deletion	+	+	+	+	barley and rice (cv. CO-39)	[282]
MGG_03123	Guy11	*mdt1*	multidrug and toxin extrusion (MATE)–family pump	fungal development and pathogenicity	deletion	NA	+	-	+	rice leaves (cultivar CO-39)	[283]

MGG_03147	70-15	*gpd2*	glycerol-3-phosphate (G-3-P) shuttle	fungal development and pathogenicity	deletion	+	+	NA	+	barley and rice (cv. CO-39)	[284]
MGG_03241	KJ201	*arrdc1*	alpha-arrestin	fungal development and pathogenicity	deletion	+	+	-	+	rice (cv. CO-39)	[285]
MGG_03313	Guy11	*vps41*	homotypic fusion and protein sorting protein complex (HOPS) protein	sexual and asexual reproduction, vacuolar morphogenesis and pathogenicity	deletion	+	+	-	+	barley (cv. Gold Promise) and rice (cv. CO39) leaves	[286]
MGG_03670	70-15	*spm1*	vacuolar serine protease	appressoria development and pathogenicity	deletion	+	+	+	+	intact and wounded barley plants	[287]
MGG_03855	Guy11	*eif3k*	eukaryotic translation initiation factor 3 (eIF3) domain containing protein	fungal development and pathogenicity	deletion	+	+	+	+	rice seedlings (cv. CO39)	[288]
MGG_04000	70-15	*rfx1*	regulatory factor X protein	fungal development and pathogenicity, chitin metabolism, cell wall integrity	deletion	+	+	+	+	rice (cv. CO-39)	[289]

MGG_04050	Guy11	*sec22*	soluble N-ethylmaleimide-sensitive factor attachment protein receptor (SNARE) proteins	fungal development and pathogenicity	deletion	+	+	+	+	rice leaves (cultivar CO-39)	[290]
MGG_04163	B157	*des1*	host-defense suppressor pathogenicity gene	fungal development and pathogenicity	RNA interference (RNAi)	+	+	+	+	rice leaves and sheath	[291]
MGG_04377	Guy11	*lrg1*	LIM (Lin-11, Isl-1 and Mec-3) protein-encoding gene	fungal development and pathogenicity	deletion	+	+	+	+	barley leaves (cv. Golden Promise)	[292]
MGG_04527	Guy11	*per1*	glycosylphosphatidylinositol (GPI)-anchored protein	fungal development and pathogenicity, cell wall integrity	deletion	+	+	+	+	rice (cv. CO-39)	[293]
MGG_04626	KV1	*mca1*	metacaspase	fungal development and pathogenicity	deletion	-	+	+	+	rice seedlings (cv. YT16)	[294]
MGG_04628	Guy11	*cyp51a*	sterol 14α-demethylase	fungal development and pathogenicity	deletion	-	+	-	+	rice leaves (cultivar CO-39)	[295]
MGG_04676	Hoku-1	*bub2*	mitotic check point protein	fungal development and pathogenicity	deletion	+	+	+	+	barley (Hordeum vulgare L. 'Nakaizumi-zairai')	[296]

MGG_05247	70-15	*mgd1*	NAD(+) dependent glutamate dehydrogenase (NAD-GDH)	fungal development and pathogenicity	deletion	+	NA	+	+	barley plants	[31]
MGG_05428	Guy11	*vam7*	soluble N-ethylmaleimide-sensitive factor attachment protein receptor (SNARE) proteins	fungal development and pathogenicity	deletion	+	+	+	+	rice leaves (cultivar CO-39)	[297]
MGG_05738	Guy11	*pax1*	LIM (Lin-11, Isl-1 and Mec-3) protein-encoding gene	fungal development and pathogenicity	deletion	+	+	+	+	barley leaves (cv. Golden Promise)	[292]
MGG_05755	Guy11	*mon1*	gene involved in sensitivity to brefeldin A and monensin	vacuolar assembly, conidiogenesis and pathogenicity	deletion	+	+	+	+	rice seedlings	[298]
MGG_05814	Guy11	*kdcdh*	family four aldehyde dehydrogenases (ALDHs), potassium-activated aldehyde dehydrogenase	fungal development and pathogenicity	T-DNA insertion and knock down	+	+	+	+	rice (cv. CO-39)	[299]
MGG_05871	B157	*pth11*	plasma membrane protein, CMEF domain-containing protein	fungal development and pathogenicity	deletion	-	+	+	+	rice seedling (cv. CO39) and barley seedling (cv. Express)	[300, 301]

MGG_06011	Guy11	*sfa1*	S-(hydroxymethyl)- glutathione dehydrogenase gene	fungal conidiation and virulence	deletion	+	+	+	+	rice (cv. CO-39)	[302]
MGG_06064	P131 and S1528	*chs7*	chitin synthase gene	fungal development and pathogenicity	deletion	NA	NA	+	+	rice leaf sheaths and barley leaves	[271]
MGG_06180	Guy11	*end3*	endocytic protein	appressorium formation and pathogenicity, F-actin assembly	deletion	-	-	+	+	rice (cv. CO39) and barley (cv. Four-arris)	[303]
MGG_06241	Guy11	*rab5a*	vacuolar protein sorting-associated protein 21	fungal development and pathogenicity	deletion	+	+	+	+	rice (cv. CO39) and barley (cv. Golden Promise)	[263]
MGG_06263	KJ201	*ypel1*	yippee-like (YPEL) gene family	fungal development and pathogenicity	deletion	+	+	+	+	rice (cv. Nakdongbyoe)	[255]
MGG_06439	B157 (field isolate, mat1-2)	*tea4*	polarity determinant	asexual development and pathogenicity	deletion	-	+	+	+	rice and barley leaves	[304]
MGG_06537	Guy11	*vps13*	vacuolar protein-sorting receptor	fungal conidiation and pathogenicity, endoplasmic reticulum (ER)-phagy	deletion	-	+	-	+	rice seedlings cv. (CO39)	[305]
MGG_06564	Guy11	*pac2*	Gti1/Pac2 family domain-containing protein	fungal development and pathogenicity	deletion	+	+	+	+	rice leaves	[306]

MGG_06843	KJ301	*abfb*	arabinofuranosidase-B protein	fungal development and pathogenicity, cell wall integrity	deletion	+	+	-	+	rice (cv. L. Jinheung)	[307]
MGG_06868	Guy11	*ilv2*	acetolactate synthase (ALS) catalytic subunit	fungal development and pathogenicity	deletion	+	+	+	+	rice and barley leaves	[262]
MGG_06898	Guy11 and KJ201	*myb1*	myeloblastosis gene	fungal development and pathogenicity	deletion	+	+	-	+	rice (cv. CO-39)	[308, 309]
MGG_07135	Guy11	*pyr5*	Ootate phosphoribosyl transferase	fungal development and virulence	deletion	+	-	+	+	rice (cv. CO-39) and barley (cv.Golden Promise)	[310]
MGG_07173	B157	*ypd1*	histidine phosphotransferase	fungal development and pathogenicity	deletion	-	+	+	+	rice (cv. CO-39)	[311]
MGG_07224	Guy11	*ilv1*	threonine deaminase	fungal conidiogenesis and pathogenesis	deletion	+	+	+	+	rice (cv. CO-39) and barley (cv. Four-arris) seedlings	[312]
MGG_07503	Guy11	*tpx1*	thioredoxin peroxidase	fungal development and pathogenicity	deletion	-	+	+	+	rice seedlings (cv. CO-39)	[266]
MGG_07599	JL0910	*prepl*	Lon/MAP1-binding protein	fungal development and pathogenicity	deletion	+	+	+	+	rice seedlings	[313]

MGG_08170	Guy11	*eif4e3*	translation initiation factor eIF4E	fungal development and pathogenicity, stress homeostasis	deletion	+	+	+	+	rice (cv. CO-39)	[314]
MGG_08203	KU80	*mbf1*	multiprotein-bridging factor 1	fungal vegetative growth, stress responses and pathogenicity	deletion	+	-	-	+	rice (cv. Nihonbare)	[315]
MGG_08345	Guy11	*cdc4*	F-box protein	fungal development and pathogenicity	deletion	+	+	+	+	barley leaves (cv. Golden Promise)	[316]
MGG_08387	YN125	*mcp1*	cytochrome P450 oxidoreductase	fungal development and pathogenicity, alleviating manganese toxin	deletion	-	+	+	+	rice seedlings (cv. LTH)	[317]
MGG_08623	Guy11	*gls2*	glycoside hydrolase	asexual/sexual development and fungal pathogenicity, cell wall integrity	deletion	+	+	+	+	rice and barley leaves	[318]
MGG_08737	B157	*fae*	feruloyl esterase	host colonisation and fungal pathogenicity	deletion	-	-	+	+	rice, wheat or barley	[319]
MGG_08829	Ku80	*tup1*	general transcriptional repressor	fungal development and virulence	deletion	+	+	+	+	rice (cv. CO-39) and barley	[320]

MGG_08843	B157	*alr2*	CorA (Metal Ion Transporter)	fungal development and pathogenicity	deletion	+	+	+	+	rice seedling (cv. HR-12)	[321]
MGG_08850	Guy11	*gti1*	Gti1/Pac2 family domain-containing protein	fungal development and pathogenicity	deletion	+	+	+	+	rice leaves	[306]
MGG_08861	P131	*cwf15*	Prp19-associated splicing factor	fungal development and pathogenicity	T-DNA insertion and deletion	+	+	+	+	rice (cv. LTH)	[322]
MGG_09022	B157	*wish*	water wettability, infection, surface sensing and hyper-conidiation	fungal development and pathogenicity, cell wall integrity	deletion	+	+	+	+	rice (cv. HR-12)	[323]
MGG_09696	Guy11	*fwd1*	F-box protein	fungal development and pathogenicity	deletion	+	+	+	+	barley leaves (cv. Golden Promise)	[316]
MGG_09902	Guy11	*capb*	β subunit of capping protein	fungal development and pathogenicity, endocytosis and actin dynamics	deletion	+	+	+	+	rice (cv. CO-39)	[324]
MGG_10422	KJ201	*afo1*	membrane-bound protein	fungal development and pathogenicity	deletion	-	+	+	+	rice seedlings (cv. Nakdongbyeo)	[325]
MGG_10492	P131 and S1528	*crc1*	carnitine–acylcarnitine carrier protein	fungal appressorium penetration, infection hyphae development and pathogenicity	deletion	-	-	+	+	rice and barley seedlings	[326]

MGG_10668	Guy11	*cgt1*	ceramide galactosyltransferase	fungal development and pathogenicity, glucosylceramide biosynthesis	deletion	+	+	+	+	barley and rice (cv. CO-39)	[282]
MGG_11141	JL0910	*map*	Magnaporthe ATP-dependent protease	appressorium formation and pathogenicity	T-DNA insertion and deletion	-	-	+	+	rice (cv. Jijing88) and barley (cv. Golden Promise)	[327]
MGG_11899	KJ201	*cdc15*	cell cycle gene	fungal development and pathogenicity	T-DNA insertion and deletion	+	+	+	+	rice (cv. Nakdongbyeo)	[328]
MGG_12005	70-15	*spf1*	P5-type ATPase	fungal development and pathogenicity	deletion	+	+	+	+	barley and rice (cv. CO39)	[329]
MGG_12818	Guy11	*capa*	α subunit of capping protein	fungal development and pathogenicity, endocytosis and actin dynamics	deletion	+	+	+	+	rice (cv. CO-39)	[324]
MGG_13013	P131 and S1528	*chs6*	chitin synthase gene	fungal development and pathogenicity	deletion	+	+	+	+	rice leaf sheaths and barley leaves	[271]
MGG_13530	KV1	*mca2*	metacaspase	fungal development and pathogenicity	deletion	-	+	+	+	rice seedlings (cv. YT16)	[294]
MGG_14008	Guy11	*ric8*	Ric8 regulator of GTP-binding protein signaling	fungal development and pathogenicity	T-DNA insertion and deletion	+	+	+	+	rice and barley leaves	[330]

MGG_14014	Guy11	*plaa*	phospholipase A2-activating protein	fungal development and pathogenicity	deletion	+	+	+	+	rice leaves (cultivar CO-39)	[331]
MGG_14279	Guy11	*gln2*	glutamine synthetase	fungal development and pathogenicity	deletion	+	+	+	+	rice (cv. CO-39)	[332]
MGG_15203	JJ88	*ae4*	bicarbonate transporter, cytomembrane and tonoplast located HCO3− transporter	fungal development and pathogenicity	deletion	-	+	+	+	rice seedlings (cv. Lijiangxintu anheigu)	[333]
MGG_16126	KJ201	*err1*	endoplasmic reticulum (ER) retention receptor 1	fungal development and pathogenicity	T-DNA insertion and deletion	+	+	+	+	rice (cv. Nakdong)	[334]
MGG_16212	70-15	*fap7*	ribosome assembly factor	fungal development and pathogenicity	deletion	+	+	+	+	rice seedlings	[335]
MGG_16213	Guy11	*myo5*	class V myosin, interacting with SEC4	fungal development and pathogenicity, endocytosis	deletion	+	+	+	+	rice leaves or barley leaves	[336]
MGG_17513	Guy11	*p5cdh*	family four aldehyde dehydrogenases (ALDHs), delta-1-pyrrorine-5-carboxylate dehydrogenase	fungal growth and pathogenicity	T-DNA insertion and knock down	+	-	-	+	rice (cv. CO-39)	[299]

The studied genes are classified into 5 categories. In Supplementary Table S1f, genes that are not discussed in detail here are ordered according to their gene codes. '+' in the table represents that the mutant phenotype is altered compared to WT, while '-' means that the phenotype remains unchanged. NA means not assessed. The secretion signals were found using 'SignalP-6.0'.
